# SynaptoTagMe, a toolkit for in vivo mapping and modulating neurotransmission at single-cell resolution

**DOI:** 10.7554/eLife.108675

**Published:** 2026-06-25

**Authors:** Andrea Cuentas-Condori, Patricia Chanabá-López, Matthew Thomas, Likui Feng, Aaron Wolfe, Peter Agoba, Matthew L Schwartz, Maximillian Brown, Margaret S Ebert, Erik Jorgensen, Cornelia I Bargmann, Daniel A Colón-Ramos

**Affiliations:** 1 Department of Neuroscience and Department of Cell Biology, Yale University School of Medicine New Haven United States; 2 Lulu and Anthony Wang Laboratory of Neural Circuits and Behavior, The Rockefeller University New York United States; 3 Howard Hughes Medical Institute and School of Biological Sciences, University of Utah Salt Lake City United States; 4 Wu Tsai Institute, Yale University New Haven United States; 5 Instituto de Neurobiología, Recinto de Ciencias Médicas, Universidad de Puerto Rico San Juan Puerto Rico; Indian Institute of Science Bangalore India; University of Cambridge United Kingdom

**Keywords:** synaptic vesicle transporter, neurotransmitters, co-transmission, synaptic terminals, monoamines, *C. elegans*

## Abstract

Understanding the organization and regulation of neurotransmission at the level of individual neurons and synapses requires tools that can track and manipulate transmitter-specific vesicles in vivo. Here, we present SynaptoTagMe, a suite of genetic tools in *Caenorhabditis elegans* to fluorescently label and conditionally ablate the vesicular transporters for glutamate, GABA, acetylcholine, and monoamines. Using a structure-guided approach informed by protein topology and evolutionary conservation, we engineered endogenously tagged versions for each transporter that maintain their physiological function while allowing for cell-specific, bright, and stable visualization. We also developed conditional knockout strains that enable targeted disruption of neurotransmitter synthesis or packaging in single neurons. We applied this toolkit to map co-expression of vesicular transporters across the *C. elegans* nervous system, revealing that over 10% of neurons exhibit co-transmission. Using the ADF sensory neuron as a case study, we demonstrate that serotonin and acetylcholine are trafficked in partially distinct vesicle pools. Our approach provides a powerful platform for mapping, monitoring, and manipulating neurotransmitter identity and use in vivo. The molecular strategies described here are likely applicable across species, offering a generalizable approach to dissect synaptic communication in vivo.

## Introduction

Understanding how the nervous system generates behavior requires tools that can resolve the molecular identity, spatial localization, and functional contribution of neurotransmitters in vivo. Neurotransmitters are the primary means by which neurons communicate, and their synthesis, packaging, and release are governed by evolutionarily conserved molecular pathways shared from *Caenorhabditis elegans* to vertebrates (Südhof, 2021). These transmitters shape the strength, kinetics (tonic vs. phasic), and polarity (excitatory vs. inhibitory) of synaptic transmission, thereby influencing how information is processed and how behavior is regulated (Crawford and Kavalali, 2015; Gouwens et al., 2020; Kamalova and Nakagawa, 2021; Liu et al., 2021). Because neurotransmitters are central to defining the functional properties of synapses, understanding their identity and dynamics is essential for interpreting circuit function. Even in organisms with complete connectomes—such as *C. elegans* (White et al., 1986) and *Drosophila melanogaster* (Scheffer et al., 2020; Seggewisse and Winding, 2024; Yi et al., 2024), anatomical connectivity alone cannot explain how neural circuits generate behaviors. To build accurate, testable models of circuit function, it is necessary to also determine which neurotransmitters are used at specific synapses and how their release is spatiotemporally organized and regulated in vivo. Yet, despite the centrality of neurotransmitters to circuit logic, the field still lacks broadly applicable tools to visualize and manipulate transmitter-specific vesicle pools with the precision needed to study their roles in intact, living animals.

Traditional approaches—such as in situ hybridization, immunohistochemistry, and transcriptomics—have been instrumental in mapping neurotransmitter identity. These methods, however, often lack cell-specific control, temporal resolution, or the ability to monitor transmitter usage dynamically within intact circuits. Moreover, neurotransmitter identity can change in response to environmental or physiological cues. For example, neurons may co-release multiple transmitters or modulate transmitter usage depending on stress, activity, or developmental stage, and these changes have consequences in animal behavior and circuit function (Chen et al., 2023; Li et al., 2024; Maddaloni et al., 2024; Sitko et al., 2025; Wu et al., 2020). Tracking and manipulating these physiological changes require new tools that allow endogenous, live imaging and functional interrogation of neurotransmitters in single neurons.

Vesicular transporters offer a strategic entry point for such investigations. These multi-pass membrane proteins package specific neurotransmitters—such as glutamate, GABA, acetylcholine, and monoamines—into synaptic vesicles and are necessary and sometimes sufficient for defining a neuron’s transmitter phenotype (Edwards, 2007). Because they are genetically encoded and highly conserved (Alfonso et al., 1993; Bellocchio et al., 1998; Chaudhry et al., 1998; Lee et al., 1999; McIntire et al., 1993; McIntire et al., 1997; Roghani et al., 1994), vesicular transporters provide a powerful molecular handle for developing generalizable tools that probe synaptic identity and function across species. Tagging these transporters can offer direct, real-time readouts of presynaptic signaling and enable manipulations that dissect the functional contribution of specific neurotransmitters in vivo (Li et al., 2020). Yet for these tools to be broadly useful, it is essential that tagging does not disrupt the localization or function of the transporter. If appropriate insertion sites can be identified and validated functionally, the evolutionary conservation of vesicular transporters suggests that such designs could serve as generalizable platforms across systems and species.

Here, we present SynaptoTagMe, a comprehensive toolkit for tracking and manipulating transmitter-specific vesicles in *C. elegans*. Using a structure-guided approach informed by predicted protein topology and sequence conservation, we engineered endogenously tagged versions of the vesicular transporters for glutamate (EAT-4/VGLUT), GABA (UNC-47/VGAT), acetylcholine (UNC-17/VAChT), and monoamines (CAT-1/VMAT). We validated in vivo that the tagged transporters retain functionality and enable bright, cell-specific imaging. In parallel, we developed conditional knockout strains that enabled spatiotemporal access to the ablation of the packaging or synthesis of specific neurotransmitters in defined neurons, allowing causal tests of neurotransmitter function at the single-cell level within behaving animals.

We applied this toolkit to identify neurons that co-express multiple vesicular transporters, revealing that 10% of *C. elegans* neurons contain the machinery for co-transmission. Focusing then on the ADF sensory neuron, we validate that ADF expresses the machinery for co-transmission of serotonin and acetylcholine. We demonstrate that serotonin and acetylcholine are packaged in partially distinct vesicle populations. Together, our observations suggest that co-transmission can be spatially organized, offering a refined view of how individual neurons diversify their signaling output in vivo. Our discoveries also highlight co-transmission as a widespread and previously underappreciated feature of nervous system organization, rather than a rare or specialized exception. Co-transmission is not unique to *C. elegans*; in *Drosophila*, the VAChT protein can be modulated in GABAergic and glutamatergic neurons by microRNAs (Chen et al., 2023); in mammals, serotonergic neurons in the dorsal raphe co-release glutamate or GABA depending on context (Li et al., 2024), while starburst amacrine cells in the retina release both acetylcholine and GABA with distinct calcium sensitivities (Lee et al., 2010; Morrie and Feller, 2015). These examples, along with our findings, underscore the evolutionary conservation of co-transmission as a mechanism for expanding the functional repertoire of single neurons.

By enabling simultaneous visualization of different transmitter-specific vesicle pools within the same neuron, our tools uncover molecular heterogeneity at individual synapses and reveal new layers of synaptic plasticity. More broadly, our findings establish a functional framework for probing neurotransmitter dynamics, synaptic architecture, and co-transmission in vivo. The strategies developed here are generalizable to other model systems and open new avenues for dissecting neural circuit logic with molecular and cellular precision.

## Results

### A systematic strategy for tagging and manipulating transmitter-specific vesicles in vivo

**Figure 1.**
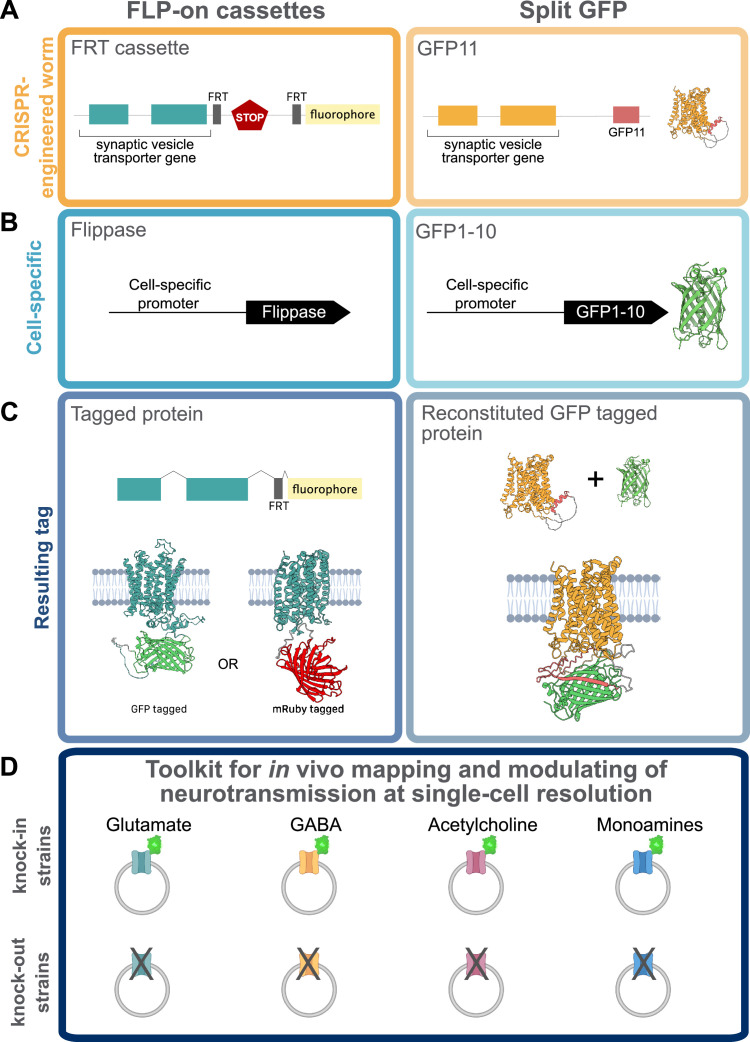
Endogenous fluorescence labeling of synaptic vesicle transporters. (Left) FLP-on and (right) split GFP strategies to endogenously label synaptic vesicle transporters. (**A**) Cartoon representation of the CRISPR-engineered worm in the endogenous locus of the target synaptic vesicle gene. (**B**) Cell-specific driver that expresses (left) Flippase or (right) GFP1-10. (**C**) Resulting tagged synaptic vesicle proteins with a (left) full-length GFP or mRuby3 or (right) by reconstituting GFP in the cell of interest. (**D**) Schematic of the resulting toolkit to label and eliminate the endogenous machinery that packages or synthesizes glutamate, GABA, acetylcholine, and monoamines. Panel D was created with BioRender.com.

All synaptic vesicle transporters are multi-pass transmembrane proteins with structural loops facing either the cytosolic or luminal space. To visualize transmitter-specific vesicle pools in vivo, we developed a suite of fluorescently tagged, functional versions of the vesicular transporters for glutamate (EAT-4/VGLUT), GABA (UNC-47/VGAT), acetylcholine (UNC-17/VAChT), and monoamines (CAT-1/VMAT) in *C. elegans*. We chose these four neurotransmitter classes because they are used by more than 90% of the neurons in *C. elegans* (Wang et al., 2024). We used a systematic design pipeline that integrated (1) protein topology predictions, (2) evolutionary conservation, and (3) structure-guided fluorophore placement to identify regions of each transporter suitable for tagging without disrupting function. These approaches were used to generate endogenous knock-in alleles with bright, cell-specific labeling through Flippase recombinase systems (Schwartz and Jorgensen, 2016) or self-assembling split-GFP tags (He et al., 2019; Figure 1). When possible, tools were developed for both green and red-based fluorophores to allow for multi-color imaging. For each transporter, we also created matched conditional knockout strains by inserting FRT-flanked cassettes to disrupt neurotransmitter packaging or synthesis in defined cells, adding to the existing cell-specific knockout tools available in the field (Huang et al., 2023; López-Cruz et al., 2019; Figure 1). To drive expression of Flippase in GABAergic and Cholinergic neurons, we inserted Flippase into the *unc-47* and *unc-17* locus after a self-cleaving 2A peptide sequence (Ahier and Jarriault, 2014), and used available Flippase drivers for Serotonergic and Dopaminergic neurons (Muñoz-Jiménez et al., 2017). Together, these new tools allow precise labeling and *loss-of-function* analysis of transmitter-specific vesicles in intact circuits and behaving animals (summarized in Table 1). All strains created in this study are accessible upon request from the *Caenorhabditis* Genetics Center (CGC), and the respective sequences are available here.

**Table 1.** Cellular, genetic, and molecular tools to probe neurotransmission in single cells.

Genotype	Purpose
**Toolkit for examining glutamatergic transmission**	
*eat-4::gfp FLP-on (syb8568)*	Cell-specific GFP knock-in (C-terminus end)
*eat-4::mRuby3 FLP-on (syb9139)*	Cell-specific mRuby3 knock-in (C-terminus end)
*eat-4 (kySi76 kySi77)*	Cell-specific knockout

**Toolkit for examining GABAergic transmission**	
*unc-47::gfp (syb6990)*	Endogenous full body GFP tagging
*unc-47::mKate2 (syb7358)*	Endogenous full body mKate2 tagging
*unc-47::gfp11 (syb7313)*	Endogenous and cell-specific GFP labeling
*unc-47::gfp11x3 (syb7849)*	Endogenous and cell-specific GFPx3 labeling
*unc-25 (syb5949 syb6275)*	Cell-specific knockout

**Toolkit for examining cholinergic transmission**	
*unc-17::mKate2 (ot907)*	Endogenous full body mKate2 tagging
*unc-17::GFP FLP-on (ola503)*	Cell-specific GFP knock-in (C-terminus end)
*unc-17::mRuby3 FLP-on (syb7882)*	Cell-specific mRuby3 knock-in (C-terminus end)
*GFP FLP-on::unc-17 (syb7251)*	Cell-specific GFP knock-in (N-terminus end)
*unc-17 (syb5779 syb5987)*	Cell-specific knockout

**Toolkit for examining monoaminergic transmission**	
*cat-1::gfp11x3 (syb7239)*	Endogenous and cell-specific GFPx3 labeling
*cat-1 (ky1101 ky1118)*	Cell-specific knockout

**Flippase drivers**	
P*eft-3*::Flippase (*sybIs9614*)	Flippase expression in all cells
*unc-47*::T2A::Flippase (*syb8125*)	Flippase expression in GABAergic neurons
*unc-17*::T2A::Flippase (*syb8059*)	Flippase expression in Cholinergic neurons
P*tph-1*::Flippase (*bqSi488*)	Flippase expression in Serotonergic neurons
P*dat-1*::Flippase (*bqSi614*)	Flippase expression in Dopaminergic neurons

### Functional labeling of glutamatergic vesicles via EAT-4/VGLUT

**Figure 2.**
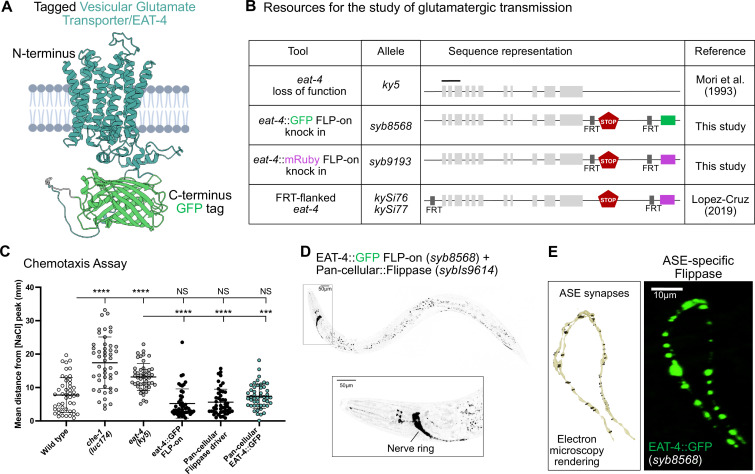
Probing glutamatergic transmission in *C*.*elegans*. (**A**) Predicted protein structure (using AlphaFold Protein Structure Database) of EAT-4 (cyan) tagged with GFP (green) (Abramson et al., 2024). The last amino acid in the C-terminus end (W576) corresponds to the tagged residue. (**B**) Schematic of the Vesicular Glutamate Transporter (VGLUT/*eat-4*) gene structure, loss of function allele (*ky5*), and endogenously tagged versions built for this study and by others. *eat-4* (*ky5*) mutants lack the first three exons. For cell-specific knock-in (KI) tools, GFP (*syb8568*) and mRuby (*syb9193*) FLP-ON cassettes (Schwartz and Jorgensen, 2016) were inserted before the STOP codon. To cell-specifically knock out (KO) *eat-4*, two FRT sites flank the coding sequence of the *eat-4* gene (*kySi76 kySi77*) (López-Cruz et al., 2019). When recombination takes place, the *eat-4* coding sequence is removed and cytosolic mCherry is inserted in-frame to be expressed as a proxy for *eat-4* sequence removal. (**C**) Mutations in the *che-1* (prevents ASE development Uchida et al., 2003) (17.42 ± 7.7 mm) or *eat-4* gene (13.15 ± 4 mm) result in disrupted migration across the salt gradient. Wild-type animals (7.76 ± 5.2 mm) migrate across the salt gradient similarly to EAT-4::GFP FLP-on (*syb*8568) animals that express (7.6 ± 3.6 mm) or not (4.9 ± 4.3 mm) flippase pan-cellularly (*sybIs9614*). Animals that express flippase in all cells (5.61 ± 3.9 mm) migrate across the salt gradient as wild-type animals. Results represent the mean distance of each worm from the salt peak, averaged across the final minute of the assay, with each dot representing a single animal. Plots are overlaid with mean ± standard deviation. Kruskal–Wallis test with Dunn’s multiple comparison post hoc test. **** represents p < 0.0001; *** represents p < 0.001 and NS means ‘not significant’. (**D**) (Top) Fluorescent image of an adult worm expressing endogenously labeled EAT-4 with GFP in all cells (P*eft-3*::Flippase). Scale bar = 50 μm. (Bottom) Zoom-in area of the head shows EAT-4 expressed predominantly in the nerve ring. Scale bar = 10 μm. (**E**) (Left) Electron microscopy rendering of ASE synapses in an L4 wild-type animal (White et al., 1986) (image generated with NeuroSC; Koonce et al., 2024). (Right) Fluorescent image of endogenously tagged EAT-4 protein cell-specifically in the ASE axons (P*flp-6*::Flippase). Scale bar = 10 μm. Figure 2—source data 1.Chemotaxis assay data graphed in panel C.

Glutamate functions as a key excitatory neurotransmitter in the nervous system, and its packaging into synaptic vesicles requires the conserved Vesicular Glutamate Transporter (VGLUT) (Bellocchio et al., 1998; Lee et al., 1999), which is sufficient to confer glutamatergic identity to a neuron. In *C. elegans*, the VGLUT homolog EAT-4 is expressed in 43 of the 118 neuronal classes cataloged (Wang et al., 2024). EAT-4/VGLUT is predicted to have 12 transmembrane domains (Figure 2A), and prior tools have allowed for cell-specific knockout of its full coding sequence (López-Cruz et al., 2019). A previously reported transgene with EAT-4/VGLUT fused to GFP demonstrated localization to synapses (Yu and Chang, 2022); here we extend this approach to an endogenously tagged allele.

**Figure 2—figure supplement 1.**
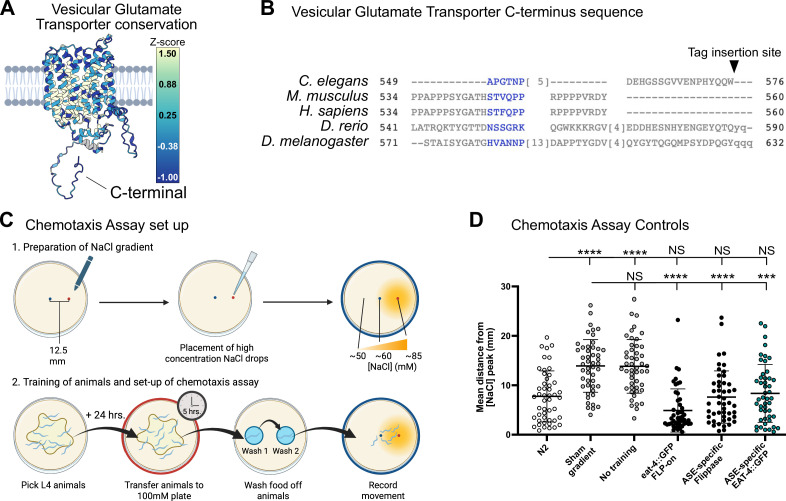
VGLUT sequence and chemotaxis assay set up. (**A**) Graphical representation of conservation of the amino acid predicted structure for the Vesicular Glutamate Transporter across common model organisms (named in panel B). Dark colors denote the least conserved region, while clear colors denote the highest conservation. Note the C-terminus end, where we introduced the GFP, is one of the darkest (least conserved) regions in the structure. (**B**) Sequence alignment of the amino acid sequences for the Vesicular Glutamate Transporter across common model organisms. Blue letters indicate conservation of the amino acid properties (basic, acid, and neutral) but not identities. Gray represents columns with gaps and no conservation. (**C**) Schematic of chemotaxis assay. (Top) A concentration gradient of NaCl (~60–85 mM) is established on assay plates (blue rim) by adding high concentration NaCl drops to the outer point of the assay plate at pre-determined times (see Methods). (Bottom) Larva stage 4 animals are picked, and 24 hr later, the young adult animals are transferred to NaCl 100 mM training plates (red rim) for 5 hr. Animals are transferred to NGM buffer drops to wash off bacteria, before being placed on the assay plates (blue rim, placement at blue dot in schematic), where animal movement away from the highest [NaCl] is recorded (toward red dot in schematic). (**D**) Displacement of trained wild-type animals on a sham gradient (13.92 ± 5.3 mm) or untrained wild-type animals on a gradient of NaCl (13.84 ± 5.4 mm). Wild-type animals (7.76 ± 5.2 mm) migrate across the salt gradient like EAT-4::GFP FLP-on (*syb8568*) animals that express (8.36 ± 5.8 mm) flippase in ASE neurons (*sybIs9606*). Animals that only express flippase in ASE neurons (7.61 ± 5.3 mm) migrate across the salt gradient similar to wild-type animals. Results represent the mean distance of each worm from the salt peak, averaged across the final minute of the assay, with each dot representing an individual animal. Plots are overlaid with mean ± standard deviation. Kruskal–Wallis test with Dunn’s multiple comparison post hoc test. **** represents p < 0.0001; *** represents p < 0.001; * represents p < 0.05; and NS means ‘not significant’. Panel C was created with BioRender.com. Figure 2—figure supplement 1—source data 1.Chemotaxis assay data graphed in panel D.

**Figure 2—figure supplement 2.**
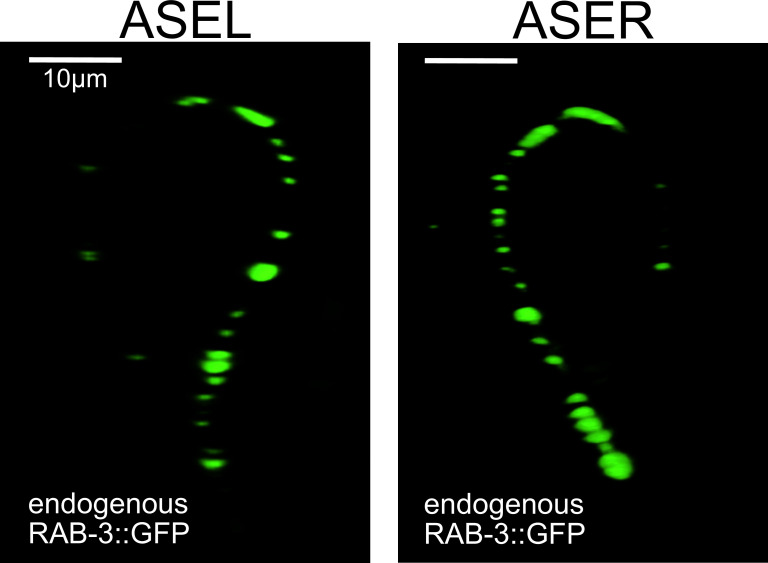
Endogenous RAB-3::GFP synaptic vesicle clusters in the axonal process of ASER and ASEL.

To generate a bright, functional reporter that reflects endogenous EAT-4/VGLUT localization in vivo, we inserted a GFP tag into the protein C-terminal cytoplasmic domain using a FLP-on cassette (Table 2) (Schwartz and Jorgensen, 2016). The C-terminus was chosen based on conservation analysis and AlphaFold structural predictions (Jumper et al., 2021; Pei and Grishin, 2001), which identified it as a cytosolic and weakly conserved region, minimizing the risk of disrupting conserved protein functions (Figure 2—figure supplement 1A, B). GFP was inserted just before the STOP codon (Figure 2B). To examine if the introduction of GFP into the endogenous EAT-4/VGLUT gene affected function, we assessed NaCl chemotaxis (Figure 2—figure supplement 1C)—an EAT-4-dependent learning behavior mediated by the ASE neurons (Figure 2—figure supplement 1C, D; Bargmann and Horvitz, 1991; Sato et al., 2021; Uchida et al., 2003). EAT-4::GFP FLP-on strains, with or without Flippase expression in all neurons or selectively in ASE neurons, displayed normal chemotaxis behavior (Figure 2C, Figure 2—figure supplement 1D), in contrast to *eat-4(ky5)* loss-of-function mutants or mutant animals with defects in chemosensory neurons, including ASE (*che-1* mutants; Figure 2C, Figure 2—figure supplement 1C, D). Our findings suggest that the tagged protein remains functional and capable of sustaining known glutamate-dependent behaviors in the organism.

**Table 2.** List of all molecular locations tested for labeling each synaptic vesicle transporter.

Gene	AA tested for fluorescent protein insertion	Functional tag?
*eat-4*	C-terminus (before STOP codon)	Yes
*unc-47*	Between E145 and N146	Yes
*unc-17*	Between AA P6-V7	Yes
*unc-17*	Between AA N242 and P243	No
*unc-17*	C-terminus (before STOP codon)	Yes

The insertion of the FLP-on cassette enables expression of EAT-4/VGLUT::GFP upon cell-specific expression on the FLP recombinase. To validate its use, we expressed pan-cellular Flippase via the *eft-3* promoter (Seydoux and Fire, 1994) in the worms engineered with the EAT-4::GFP FLP-on cassette. We observed bright EAT-4::GFP signal throughout the nervous system, especially in the nerve ring and sensory neurons (Figure 2D), consistent with earlier transcriptional reporters of the *eat-4* gene (Lee et al., 1999; Serrano-Saiz et al., 2013). Moreover, when Flippase was driven specifically in ASE neurons (using the ASE-specific promoter, P*flp-6*), we observed punctate labeling along ASE axons, matching the distribution of presynaptic sites identified by serial electron microscopy (Figure 2E), and cataloged in NeuroSC (https://neurosc.net/) (Koonce et al., 2024; White et al., 1986). Consistent with this, we observed that endogenous GFP::RAB-3 in ASE similarly localizes in a punctate pattern along the axons in a pattern that is reminiscent of that seen for endogenous EAT-4::GFP (Figure 2E, Figure 2—figure supplement 2).

To expand the utility of this tool for multicolor imaging, we also generated a red-shifted FLP-on reporter for the *eat-4/VGLUT* gene by inserting mRuby3 (Bajar et al., 2016) with a *C. elegans*-optimized sequence at the same C-terminal site (Figure 1B). These spectrally distinct reporters, when combined with the previously developed *eat-4* conditional knockout (López-Cruz et al., 2019), provide a comprehensive toolkit for dissecting glutamatergic transmission in a cell-specific manner in vivo.

### Cell-specific imaging and silencing of GABAergic neurotransmission

**Figure 3.**
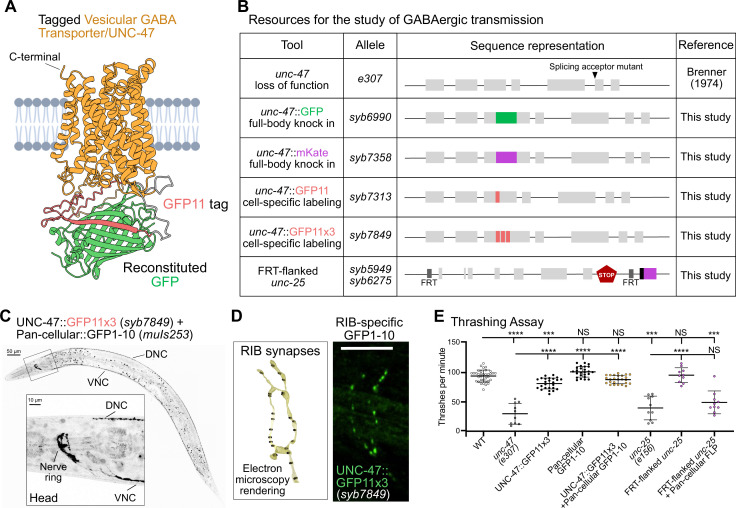
Probing GABAergic transmission in *C*. *elegans.* (**A**) UNC-47 predicted protein structure from AlphaFold Protein Structure Database. GFP11 tag (red) was added between amino acids E145 and N146. Complementary GFP1-10 (green) was modeled as bound to GFP11. (**B**) Schematics of the Vesicular GABA Transporter (VGAT/*unc-47*) loss of function allele and endogenously tagged versions built for this study. *unc-47(e307)* mutant animals have a single base pair substitution (G to A in the first nucleotide of exon 6) that results in a splicing acceptor mutant. Fluorescent tags GFP (*syb6990*) and mKate2 (*syb7358*) were inserted between amino acids E145 and N146. For cell-specific endogenous labeling, UNC-47 was tagged with one (*syb7313*) or three copies (*syb7849*) of GFP11. To silence GABA transmission, we flanked the Glutamic Acid Decarboxylase/*unc-25* coding sequence with two FRT sites (*syb5949, syb6275*). Upon recombination, the *unc-25* coding sequence is removed and nuclear (black) mCherry (purple) is designed to be in-frame and expressed as a proxy for *unc-25* sequence removal. (**C**) (Top) Fluorescent image of an adult worm expressing endogenously labeled UNC-47 with reconstituted split-GFP in all cells (P*eft-3*::GFP1-10). Scale bar = 50 μm. (Bottom) Zoom-in area of the head shows UNC-47 expressed in the nerve ring and nerve cords. Scale bar = 10 μm. (**D**) (Left) Electron microscopy renderings of RIB synapses in an L4 wild-type animal (White et al., 1986) (image generated with NeuroSC[https://neurosc.net/]; Koonce et al., 2024). (Right) RIB-specific UNC-47 puncta (green) in vivo using the UNC-47:GFP11 with reconstituted split-GFP in RIB (P*sto-3b*::GFP1-10). Scale bar = 10 μm. (**E**) *unc-47(e307*) mutant animals thrash significantly less per minute (30.8 ± 17) than wild-type animals (94.5 ± 10). Animals with non-reconstituted UNC-47::GFP11X3 (82.2 ± 9) (*syb7849*) thrash slightly less than wild-type animals (94.5 ± 10), while animals that express pan-cellular GFP1-10 (100.5 ± 9) (*muIs253*) are no different than wild-type animals. UNC-47::GFP11x3 animals that express Pan-cellular::GFP1-10 (with reconstituted GFP) (88.6 ± 7) thrash similarly to wild-type animals. FRT-flanked *unc-25* animals (*syb5949*, *syb6275*) that do not express Flippase (95.8 ± 12) thrash similarly to wild-type animals (91 ± 8). Cell-specific *unc-25* knockout animals (*syb5949*, *syb6275*) that express Flippase in every neuron (*bqSi506*) (49.36 ± 19) thrash to the same extent as *unc-25* (*e156*) mutant animals (40.2 ± 19). Plots are overlaid with mean ± standard deviation. Kruskal–Wallis test with Dunn’s multiple comparison post hoc test. **** represents p < 0.0001; *** represents p < 0.001; and NS means ‘not significant’. Figure 3—source data 1.Chemotaxis assay data graphed in panel E.

GABAergic neurons package GABA into synaptic vesicles via the conserved vesicular GABA transporter VGAT (Chaudhry et al., 1998; McIntire et al., 1997; Mclntire et al., 1993). In *C. elegans*, the VGAT homolog UNC-47 is expressed in 11 of the 118 neuronal classes (Gendrel et al., 2016; Wang et al., 2024). Based on in vivo data, the N-terminus of VGAT is cytoplasmic while the C-terminus is luminal (Martens et al., 2008). The N-terminus contains dileucine motifs critical for proper trafficking (Santos et al., 2013), and to preserve transporter function we focused on tagging long cytoplasmic loops. Structural predictions from AlphaFold indicate that UNC-47 has 11 transmembrane domains (Figure 3A), and we identified the cytosolic loop between transmembrane domains 2 and 3—a long (13 amino acids) region (Figure 3—figure supplement 1A, B)—as an optimal tagging site (Table 2).

**Figure 3—figure supplement 1.**
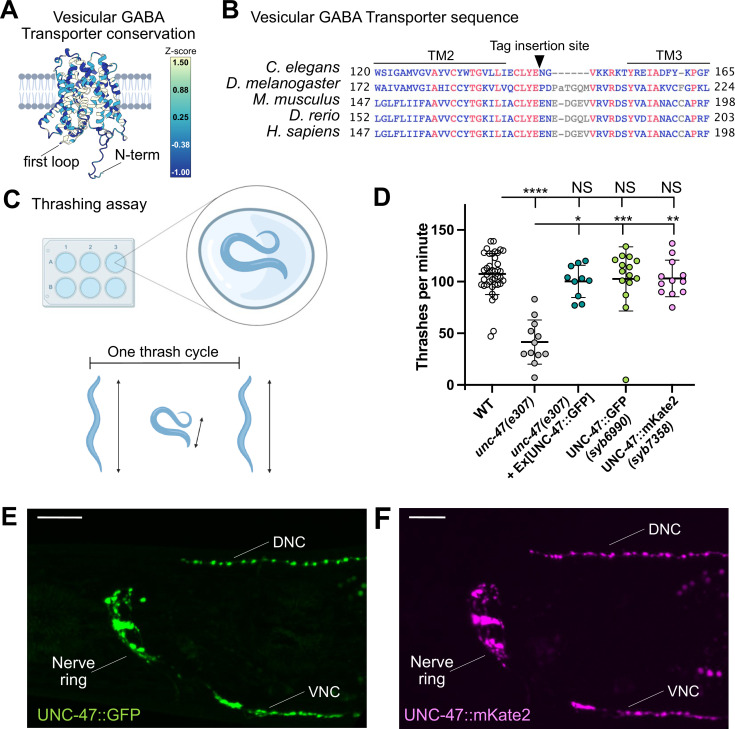
VGAT sequence and swimming assay set up. (**A**) Graphical representation of conservation of the Vesicular GABA Transporter amino acid predicted structure across common model organisms (named in Figure 2B). Dark colors denote the least conserved region, while clear colors denote the highest conservation. (**B**) Sequence alignment of the cytosolic loop between transmembrane domains 2 and 3 of the vesicular GABA transporter across common model organisms. Red letters indicate highly conserved columns (conservation of amino acid identity), and blue letters indicate conservation of the amino acid properties (basic, acid, and neutral) but not identities. Gray represents columns with gaps. (**C**) Schematic of thrashing assay. ‘One thrash cycle’ is scored when the animal bends as indicated in the schematic (from elongated-to-bent-to-elongated). We measured the number of thrashes per minute. (**D**) *unc-47(e307)* mutant animals thrash significantly less (41.5 ± 21.35) than wild-type animals (109.2 ± 23), while over-expression of UNC-47::GFP (100.2 ± 16), CRISPR knock-in UNC-47::GFP (107.9 ± 29), and UNC-47::mKate2 (103.1 ± 18) CRISPR-tagged animals thrash similarly to wild-type animals. Plots are overlaid with mean ± standard deviation. Kruskal–Wallis test with Dunn’s multiple comparison post hoc test. **** represents p < 0.0001; *** represents p < 0.001; ** represents p < 0.01; * represents p < 0.05; and NS means ‘not significant’. Fluorescent image of endogenously tagged (**E**) UNC-47::GFP (*syb6990*) and (**F**) UNC-47::mKate2 (*syb7358*). DNC = dorsal nerve cord, VNC = ventral nerve cord. Scale bar = 10 μm. Panel C was created with BioRender.com. Figure 3—figure supplement 1—source data 1.Thrashing assay data graphed in panel D.

**Figure 3—figure supplement 2.**
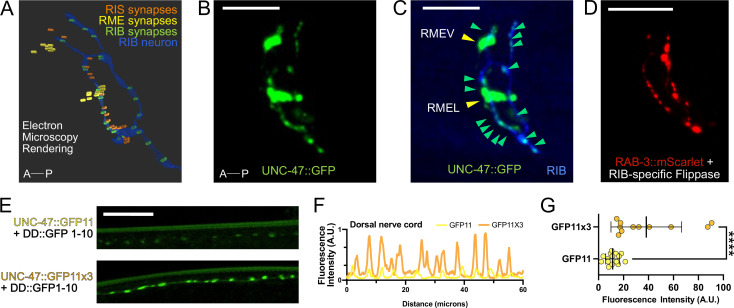
Cell-specific labeling of UNC-47 in vivo. (**A**) All presynaptic sites from known GABAergic neurons in the *C. elegans* nerve ring (RIS, RME, and RIB), according to 3D electron microscopy of a Larva stage 4 (L4) wild-type animal (White et al., 1986) (image generated with NeuroSC [https://neurosc.net/]; Koonce et al., 2024). (**B**) 3D-rendering of UNC-47::GFP (green) puncta in the *C. elegans* nerve ring. A–P denotes anterior–posterior axis. Scale bar = 10 μm. (**C**) Addition of RIB::BFP into Figure 3B. Green arrowheads point to UNC-47::GFP puncta that overlap with RIB::BFP. Yellow arrowheads point to the synapses we interpret, based on our labeling and distribution, to belong to the RME neurons. (**D**) Endogenous mScarlet::RAB-3 in RIB highlight the characteristic shape of the RIB axon. (**E**) In vivo reconstitution of UNC-47::GFP11x3 in DD neurons when tagged with (Top) one or (Bottom) three copies of GFP11 (P*flp-13*::GFP1-10) (He et al., 2019). (**F**) Line scans and (**G**) quantification of reconstituted GFP fluorescence intensity with one (11.3 ± 6) or three copies (38.3 ± 28) of GFP11 in DD neurons. Scale bar = 10 μm. Plots are overlaid with mean ± standard deviation. Mann–Whitney test. **** represents p < 0.0001. Figure 3—figure supplement 2—source data 1.Fluorescence data graphed in panel G.

Within this loop, AlphaFold predicts two beta-sheet regions with high confidence. We inserted GFP between amino acids E145 and N146, immediately following the first predicted beta sheet, to avoid disrupting secondary structures (Figure 3, Figure 3—figure supplement 1). To assess functionality of the newly engineered UNC-47/VGAT::GFP strain, we performed thrashing assays on *unc-47 (e307)* mutants, which show impaired locomotion due to loss of GABA signaling at neuromuscular junctions (McIntire et al., 1993). Expression of UNC-47::GFP from an extrachromosomal array rescued the thrashing defect to wild-type levels (Figure 3—figure supplement 1C, D), as expected. We next generated endogenous knock-ins of GFP and mKate2 at the same site but in the endogenous *unc-47* locus, and observed wild-type locomotion for these strains, consistent with the insertion of the fluorophores not affecting endogenous function of the transporter (Figure 3—figure supplement 1D). These strains showed bright, punctate fluorescence in the nerve ring and along the dorsal and ventral nerve cords (Figure 3—figure supplement 1E, F), consistent with previously reported *unc-47* expression patterns (McIntire et al., 1997). Together, these results demonstrate that inserting a fluorescent protein at position E145 results in a functionally tagged UNC-47/VGAT reporter that enables endogenous visualization of the protein.

To enable in vivo visualization of GABAergic vesicles in single cells, we next generated two UNC-47::splitGFP alleles by inserting either one or three tandem copies of GFP11 at the E145 position (Figure 3B). We used the splitGFP approach to avoid disruptions of the protein structures due to the introduction of the FRT-cassettes at an internal sequence site. By leveraging the self-assembling property of the GFP beta barrel, knock-in of the eleventh beta strand (GFP11) results in labeling of a protein that is only visible when the complementary GFP1-10 is expressed in the same cell. This property results in a combinatorial labeling strategy, in which cell-specific labeling is achieved only in those cells that express both the GFP11 and the GFP 1–10 (He et al., 2019). To validate these tools, we first achieved pan-cellular expression of GFP1-10 (*eft-3* promoter) (Seydoux and Fire, 1994) in animals carrying the UNC-47::GFP11x3 (*syb7849*) allele. We observed GABAergic synapses throughout the nerve ring and nerve cords (Figure 3C), similar to full-body knock-in strains (Figure 3—figure supplement 1E, F). To then visualize GABAergic vesicles in subsets of cells, we expressed GFP1-10 in the GABAergic DD motor neurons using the *flp-13* promoter and in animals carrying the GFP11 (*syb7313*) or UNC-47::GFP11x3 (*syb7849*) alleles. We observed punctate reconstituted signal in the dorsal nerve cord of both GFP11 and GFP11x3 strains, consistent with the known distribution of DD synapses (Figure 3—figure supplement 2E). The triple GFP11 version produced significantly brighter signal (Figure 3—figure supplement 2E–G), in line with reports of enhanced fluorescence from multimerized tags (He et al., 2019).

To then visualize GABAergic synapses in single cells, we expressed GFP1-10 under an RIB-interneuron, cell-specific promoter. We selected RIB because it is a GABAergic interneuron embedded in the nerve ring and proximal to three other GABAergic interneurons with overlapping neurites that impede visualization of RIB-specific synapses in vivo when using traditional approaches (Figure 3—figure supplement 2A–C). Reconstituted fluorescence using our tools enabled visualization of RIB-specific synapses, and the observed synaptic pattern was consistent with the pattern expected from electron microscopy reconstructions (Figure 3D; Koonce et al., 2024; White et al., 1986) (https://neurosc.net/) and from expression of endogenous mScarlet::RAB-3 in RIB neurons (Figure 3—figure supplement 2D). These findings underscore the value of the tool in labeling GABAergic synapses in individual cells in vivo.

To functionally dissect GABAergic transmission, we developed a conditional knockout of *unc-25*, the gene encoding Glutamic Acid Decarboxylase (GAD), which catalyzes GABA synthesis. We decided to target *unc-25*/GAD because it results in the elimination of GABA from neurons (Gendrel et al., 2016). We flanked the *unc-25* coding sequence with FRT sites and inserted a nuclear mCherry reporter downstream to indicate successful recombination (Figure 3B). We then validated the tool by using thrashing assays. Consistent with previous findings, *unc-25* null mutants show severely reduced locomotion in the thrashing assays (McIntire et al., 1993; Figure 3E). We observed that animals carrying the conditional knockout allele behaved normally in the absence of Flippase, but that pan-cellular Flippase expression in all GABAergic neurons (*unc-47* promoter), which is expected to result in loss of GABAergic neurotransmission, phenocopied the thrashing defect of *unc-25* mutants (Figure 3E). These results confirm that this tool effectively eliminates *unc-25/*GAD activity, with the capacity to be activated in a cell-specific manner and allows investigation of how GABAergic transmission contributes to neural function and behavior.

### Functional labeling of cholinergic vesicles via UNC-17/VAChT

The cholinergic identity of neurons is defined by the expression of a conserved gene locus, shared from nematodes to vertebrates, that includes both the acetylcholine synthesis enzyme Choline Acetyltransferase (ChAT) and the Vesicular Acetylcholine Transporter (VAChT) (Eiden, 1998). In *C. elegans*, the VAChT homolog UNC-17 is expressed in 57 of the 118 neuronal classes (Wang et al., 2024). Based on prior studies, both the N- and C-termini of VAChT face the cytosol and contain regulatory motifs important for trafficking (Fei et al., 2008). AlphaFold predicts UNC-17 has 12 transmembrane domains, but most cytosolic loops are very short (<5 amino acids), except for the third cytoplasmic loop, which contains 28 amino acids and exhibits relatively high sequence conservation (Figure 4—figure supplement 1A, B).

**Figure 4.**
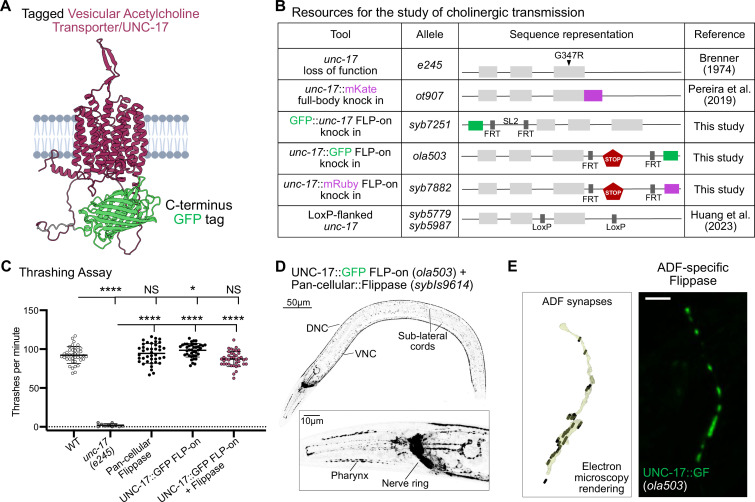
Probing cholinergic transmission in *C*. *elegans.* (**A**) Predicted UNC-17 protein structure (magenta) tagged with GFP (green) on the last amino acid in the C-terminus end (W532). (**B**) Schematics of the Vesicular Acetylcholine Transporter (VACHT/*unc-17*) loss-of-function allele and endogenously tagged versions tested in this study (and built by others). *unc-17(e245)* mutant animals have a single base pair substitution in the third exon which leads to an amino acid change (arrowhead). Full-body knock-in animal labels UNC-17 with mKate (*ot907*). For cell-specific labeling, the N-terminus GFP FLP-ON cassette (*syb7251*) was inserted between amino acids P6 and V7 (see Methods). Similarly, the C-terminus GFP FLP-ON cassette (*ola503*) and mRuby FLP-on cassette (*syb7882*) were inserted before the STOP codon. (**C**) *unc-17(e245)* mutant animals (1.9 ± 2) thrash significantly less than wild-type animals (93 ± 10). UNC-17::GFP FLP-on (*ola503*) animals that express pan-cellular::Flippase (88 ± 1) (*sybIs9614*) or animals that only express pan-cellular::Flippase (94.8 ± 12) are indistinguishable from wild-type animals in their thrashing behavior. UNC-17::GFP FLP-on animals (98.7 ± 8) thrash significantly more than wild-type animals. Mean ± standard deviation. Brown–Forsythe ANOVA test with Dunnett’s T3 multiple comparisons post hoc test. **** represents p < 0.0001; * represents p < 0.05; and NS means ‘not significant’. (**D**) (Top) Fluorescent image of an adult worm expressing endogenously labeled UNC-17::GFP in all cells (P*eft-3*::Flippase). Scale bar = 50 μm. (Bottom) Zoom-in area of the head shows UNC-17 expressed in the nerve ring, nerve cords and sub-lateral cords. DNC = dorsal nerve cord, VNC = ventral nerve cord. Scale bar = 10 μm. (**E**) (Left) Electron microscopy rendering of ADF synapses in an L4 wild-type animal (White et al., 1986) (image generated with NeuroSC [https://neurosc.net/]; Koonce et al., 2024). (Right) Fluorescence image of endogenously tagged UNC-17::GFP protein specifically in the ADF neuron. Scale bar = 10 μm. Figure 4—source data 1.Thrashing assay data graphed in panel C.

To determine suitable tagging sites for imaging UNC-17 without disrupting function, we tested three locations (Table 2): the conserved third cytosolic loop (site 1), the N-terminus (site 2), and the C-terminus (site 3) (Figure 4—figure supplement 1B, C, E). We used a thrashing assay to assess function, as *unc-17(e245*) null mutants fail to thrash in liquid and are rescued by the re-expression of untagged UNC-17 under its own promoter (Figure 4—figure supplement 1F). We generated transgenic strains with a rescue array containing the *unc-17* gene with GFP inserted into these three sites. We observed that GFP insertion into site 1 failed to rescue the phenotype, suggesting disruption of UNC-17 function. In contrast, tagging either the N-terminus or C-terminus restored normal thrashing (Figure 4—figure supplement 1E, F), indicating that these positions tolerate modification. Informed by these rescue experiments, we then generated two FLP-on conditional knockout alleles (Schwartz and Jorgensen, 2016) with GFP inserted at either the N-terminus (*syb7251*) or C-terminus (*ola503*) (Figure 4A, B). To test whether the FLP-on cassettes affected protein function, we examined behavior before Flippase expression and found that both alleles behaved like wild-type animals (Figure 4—figure supplement 1F). Because splicing regulatory elements are located near the 5′ end of the *unc-17* gene and are required for successful splicing of the cholinergic locus (both *unc-17* and *cha-1* transcripts) (Mathews et al., 2015), we proceeded with the C-terminally tagged *ola503* allele, which leaves the 5′ region intact. We tested whether pan-cellular expression of Flippase in this strain impairs behavior. Animals with global GFP-tagged UNC-17 showed wild-type thrashing in liquid (Figure 4C), confirming that the tagged transporter is functional and validating this approach for cell-specific labeling of cholinergic vesicle pools.

**Figure 4—figure supplement 1.**
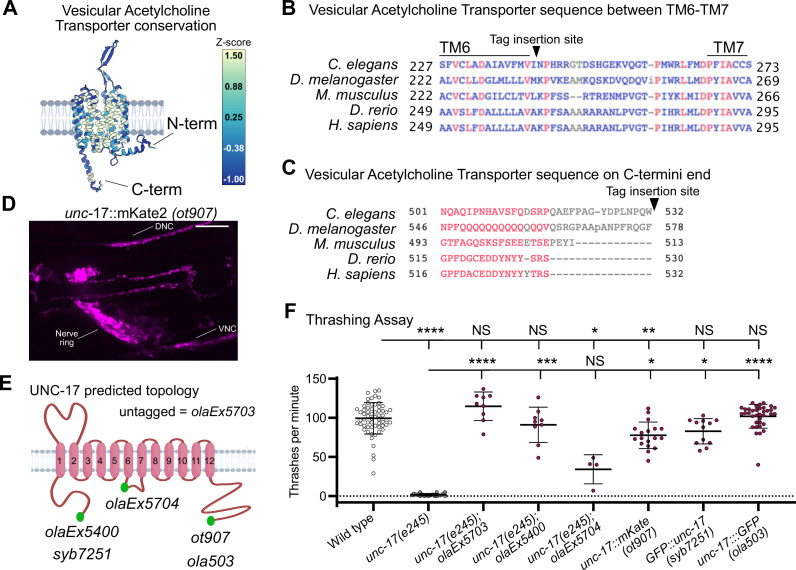
VAChT sequence and location of tag insertions. (**A**) Graphical representation of the amino acid conservation of the Vesicular Acetylcholine Transporter (VAChT) across common model organisms. Dark colors denote the least conserved region, while clear colors denote the highest conservation. Note the C-terminus end is one of the darkest regions in the structure, where the fluorophore was added. (**B, C**) Amino acid sequence alignment of VAChT across model organisms. Black arrowhead points to insertion site of fluorescent tag. (**B**) Sequences between transmembrane domains 6 and 7, which was tagged in *olaEx5704* array (See panel E). (**C**) Sequence at the C-terminus end. Red letters indicate highly conserved columns (exact conservation of the amino acid identity), and blue indicates conservation of the amino acid properties (basic, acid, and neutral) but not identities. Gray represents areas of no conservation. (**D**) Fluorescence image of CRISPR-tagged UNC-17::mKate2 (*ot907*) animal. Scale bar = 10 μm. (**E**) Schematic of UNC-17 predicted topology (magenta) along the membrane (gray). Green represents the locations of fluorescent tags tested for thrashing assays. Alleles, either extrachromosomal arrays expressed in *unc-17* (*e245*) mutants or CRISPR knock-in strains in the *unc-17* locus, are listed based on where the fluorescent tag was inserted. (**F**) *unc-17(e245)* mutant animals barely swim (1.7 ± 2). Over-expression of untagged P*unc-17*::UNC-17 (*olaEx5703*) (114.8 ± 18) and N-terminus-tagged UNC-17::GFP (*olaEx5400*) (91.1 ± 22) swim like wild-type animals (99.4 ± 20). GFP-tag between TM6-7 (*olaEx5704*) (34.3 ± 19) or CRISPR-insertion of C-terminus mKate2 tag (*ot907*) (77.7 ± 17) leads to reduced swimming behavior when compared to wild-type animals. Insertion of GFP-FLP-on cassette at the N-terminus end (s*yb7251*) (82.9 ± 16) or insertion of the GFP-FLP-on cassette at the C-terminus (*ola503*) (101.9 ± 16) thrash as well as wild-type animals (109 ± 25). We are unsure as to the phenotypic differences of *ola503* allele and *ot907* in our thrashing assays but note that in addition to the use of different fluorescent proteins, each allele also employs distinct linker sequences between UNC-17 and the fluorescent protein (Figure 4–Supplement 1). *syb7882* allele, which employs mRuby3 with a similar linker to *ola503*, also does not display defects in the thrashing assay (Figure 4—figure supplement 2). Mean ± standard deviation. Kruskal–Wallis test with Dunn’s multiple comparison post hoc test. **** represents p < 0.0001; ** represents p < 0.01; * represents p < 0.05; and NS means ‘not significant’. Figure 4—figure supplement 1—source data 1.Thrashing assay data graphed in panel F.

**Figure 4—figure supplement 2.**
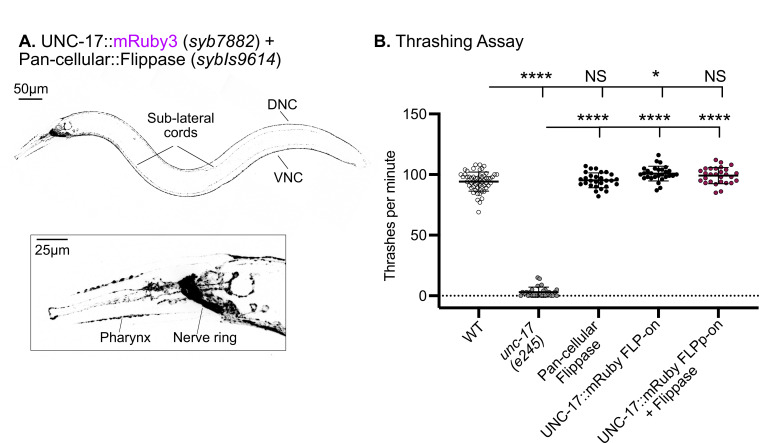
Functional validation of *unc-17*::mRuby3 (*syb7882*) allele. (**A**) (Top) Fluorescent image of an adult worm expressing endogenously labeled UNC-17::mRuby3 in all cells (P*eft-3*::Flippase). Scale bar = 50 μm. (Bottom) Zoom-in area of the head shows UNC-17 expressed in the nerve ring. DNC = dorsal nerve cord, VNC = ventral nerve cord. Scale bar = 25 μm. (**B**) *unc-17(e245)* mutant animals (3 ± 4.1) thrash significantly less than wild-type animals (94 ± 7.8). UNC-17::mRuby3 FLP-on (*syb7882*) animals that express pan-cellular::Flippase (99 ± 6.6) (*sybIs9614*) or animals that only express pan-cellular::Flippase (95 ± 6.2) are indistinguishable from wild-type animals in their thrashing behavior. UNC-17::mRuby FLP-on animals (100 ± 6.1) thrash significantly more than wild-type animals. Mean ± standard deviation. Kruskal–Wallis ANOVA test with Dunn’s multiple comparisons post hoc test. **** represents p < 0.0001; * represents p < 0.05; and NS means ‘not significant’. Figure 4—figure supplement 2—source data 1.Thrashing assay data graphed in panel B.

At the cellular level, GFP labeling of UNC-17/VAChT using the *ola503* allele and pan-cellular Flippase expression (driven by the *eft-3* promoter) (Seydoux and Fire, 1994) resulted in fluorescence in the nerve ring, dorsal cord, ventral cord, and sublateral cords (Figure 4D). This expression pattern matched that of a full-body mKate2 knock-in of endogenous UNC-17/VAChT (Figure 4—figure supplement 1D; Pereira et al., 2019) and was consistent with prior transcriptional reporters (Pereira et al., 2015) and anti-UNC-17 antibody staining (Duerr et al., 2008). The *ola503* allele also enables cell-specific labeling. When Flippase was expressed specifically in ADF neurons using the P*srh-142* promoter (Maicas et al., 2021), we observed UNC-17::GFP puncta along the ADF axon (Figure 4E). This punctate pattern aligned with known ADF presynaptic sites from electron microscopy reconstructions of L4 animals (https://neurosc.net/) (Koonce et al., 2024; White et al., 1986). Together, these results demonstrate that the UNC-17::GFP FLP-on allele provides a reliable tool to label cholinergic vesicles in individual neurons in vivo, and that this labeling does not affect function in our assays. To enable multicolor imaging, we generated a red, fluorescent version of the UNC-17/VAChT tool by replacing GFP with *C. elegans*-optimized mRuby3 (Figure 4B). This mRuby-tagged allele performs as wild-type animals in a thrashing assay and labels synaptic structures along the nerve ring and nerve cords (Figure 4—figure supplement 2A, B). Combined with the previously described cell-specific *unc-17* knockout strain (Huang et al., 2023; Figure 4B), these tools allow precise tracking and manipulation of cholinergic neurotransmission in vivo.

### Probing monoaminergic transmission

**Figure 5.**
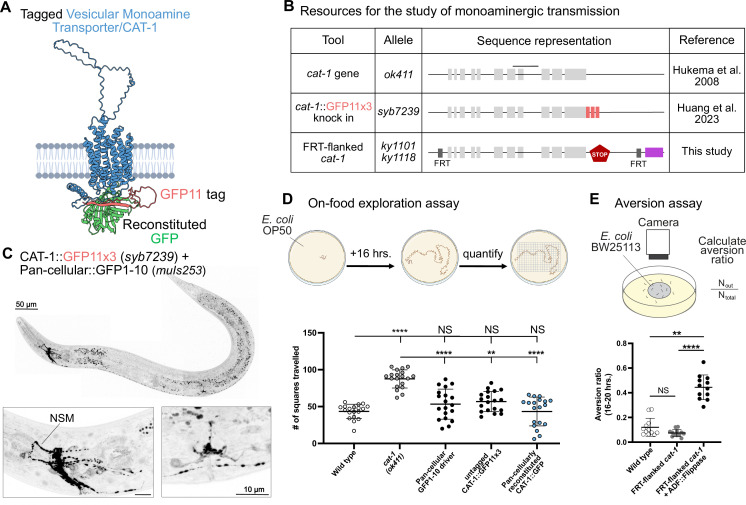
Probing Monoaminergic transmission in *C*. *elegans.* (**A**) Predicted CAT-1 protein structure (blue) labeled with GFP11 (red) at C-terminus end (E145). Complementary GFP1-10 (green) was modeled with bound GFP11. (**B**) Schematic of the Vesicular Monoamine Transporter (VMAT/*cat-1*) loss-of-function allele and endogenously tagged versions used to monitor monoaminergic transmission. *cat-1(ok411*) mutant animals have a deletion that spans exons 7 and 8 (black line) of the *cat-1* gene. For cell-specific labeling, three copies of GFP11 (*syb7239*) were inserted before the STOP codon. For cell-specific silencing of *cat-1* activity, two FRT sites (*ky1101 ky1118*) flank the coding sequence of the *cat-1* gene. Upon recombination, expression of cytosolic mCherry (magenta) is used as a proxy for deletion of the *cat-1* coding sequence. (**C**) (Top) Fluorescence image of reconstituted CAT-1::GFP11x3 with expression of complementary GFP1-10 in the whole animal (P*eft-3*::GFP1-10). Scale bar = 50 μm. (Bottom) Zoom-in of the (Left) head and (Right) vulva area of an adult worm shows CAT-1 expressed in the nerve ring and pharyngeal neurons as well as in the vulva. Scale bar = 10 μm. (**D**) (Top) A well-fed day-1 adult animal is placed on NGM plates covered with a thin layer of bacteria, allowed to roam for 16 hr and the number of squares traveled was recorded. (Bottom) Wild-type animals (43.6 ± 10) roam less than *cat-1(ok411*) mutants (87.5±12). CAT-1::GFP11x3 (*syb7239*) animals with GFP reconstituted (43.3 ± 20) (or not (56.8 ± 14)) roam similar to wild-type animals. Animals with pan-cellular expression of GFP1-10 (*muIs253*) (53.3 ± 20) also roam like wild-type animals. (**E**) (Top) A well-fed day-1 adult animal is placed on NGM plates covered with a thin layer of *E. coli*, allowed to roam for 20 hr and the aversion ratio was calculated. (Bottom) Wild-type animals (0.12±0.07) display less aversion to *E. coli* lawns than ADF-specific *cat-1* conditional KO animals (*ky1101 ky1118; syb9159*) (0.4 ± 0.1), consistent with previous reports using serotonin-depletion mutants (Feng et al., 2025). *cat-1* conditional KO animals that do not express Flippase in ADF neurons (0.07 ± 0.03) are indistinguishable from wild-type. Mean ± standard deviation. Kruskal–Wallis test with Dunn’s multiple comparison post hoc test. **** represents p < 0.0001; ** represents p < 0.01; * represents p < 0.05; and NS means ‘not significant’. Figure 5—source data 1.On-food exploration assay data graphed in panel D. Figure 5—source data 2.Aversion assay data graphed in panel E.

Monoamines such as serotonin, dopamine, norepinephrine, epinephrine, octopamine, and tyramine are transported into vesicles by the conserved Vesicular Monoamine transporter (Duerr et al., 1999; Erickson et al., 1992). In *C. elegans*, the VMAT homolog CAT-1 is expressed in 16 of the 118 neuronal classes (Wang et al., 2024). Recently, CAT-1 was tagged at its C-terminus with a split GFP11x3 reporter (Figure 5A, B; Huang et al., 2023). When GFP is reconstituted pan-cellularly, this fusion produces a punctate signal enriched in the nerve ring—including the characteristic and well-known serotonergic neuron NSM—and in serotonergic neurons that are part of the reproductive organs (Figure 5C).

To determine whether the CAT-1::GFP11x3 fusion maintains protein function, we used a behavioral assay based on the role of serotonin to prevent animal exploration on a lawn of bacteria (Flavell et al., 2013). Since serotonin is packaged into vesicles by CAT-1/VMAT, similar to mutants of serotonin production (Flavell et al., 2013), *cat-1* mutants display increased exploration behavior compared to wild-type animals (Figure 5D). We found that animals with reconstituted CAT-1::GFP11x3 explore bacterial lawns at levels comparable to wild-type (Figure 5D), indicating that the GFP11x3 tag nor its reconstitution impair CAT-1 function. To complement this tool, we also developed a cell-specific *cat-1* knockout allele in which the full coding sequence is excised upon Flippase expression (Figure 5B). Removal of serotonin production (*tph-1*) specifically from ADF neurons results in increased aversion from wild-type *E. coli* bacteria lawns (Feng et al., 2025). Consistent with this, cell-specific KO of *cat-1* in ADF neurons (via expression of ADF::Flippase) results in increased *E. coli* aversion (Figure 5E). Together, these tools now enable the cell-specific tracking and silencing of monoaminergic synapses in living animals.

### In vivo identification of co-transmitter neurons

**Figure 6.**
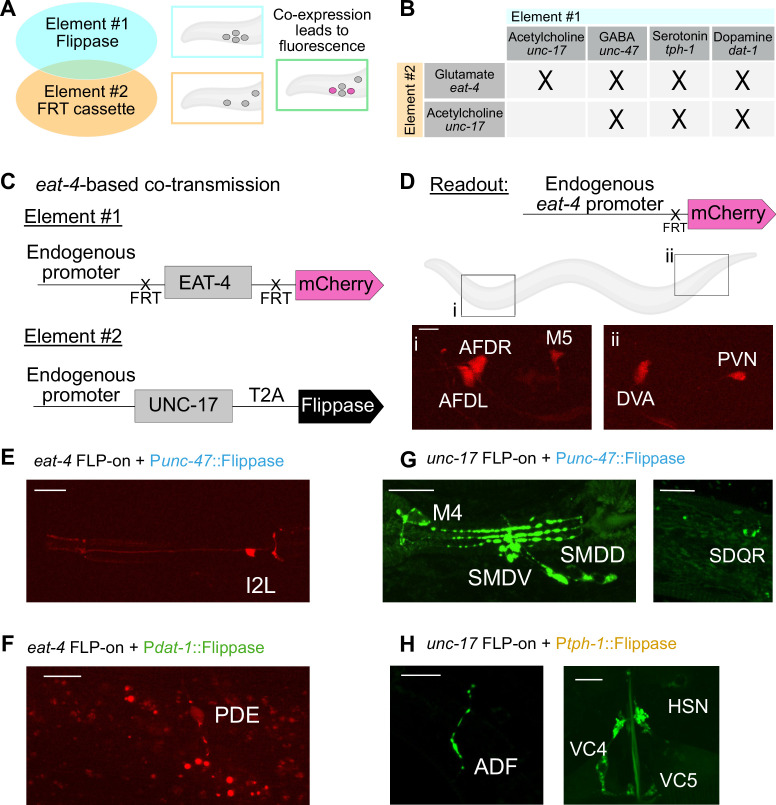
Mapping of co-transmitter neurons in the *C*. *elegans* nervous system. (**A**) Schematic of the approach used to find co-expression of two vesicular transporters in the same cells. Flippase drivers (Element #1, blue) and Flippase-dependent cassettes that result in fluorescence (Element #2, orange) are used. Magenta cells are seen when both elements are co-expressed (in green box). (**B**) Strategy to track the co-expression of the vesicular glutamate and acetylcholine transporter in combination with the 4 most common neurotransmitters in *C. elegans*: Acetylcholine, GABA, serotonin, and dopamine. ‘Elements #1’ or ‘Element #2’ refers to the genetic elements in the schematic in A. (**C, D**) Specific example of the genetic strategy outlined in A, for the vesicular transporters of glutamate (EAT-4) and acetylcholine (UNC-17). (**C**) We repurposed the *eat-4* conditional KO strain (*kySi76 kySi77*) (Figure 1B; López-Cruz et al., 2019) in which the *eat-4* gene coding sequence is flanked by two FRT sites and followed by cytosolic mCherry (Element #1). We crossed this line with a strain that has an endogenously inserted self-cleaving peptide sequence (T2A) followed by Flippase before the STOP codon in the *unc-17* gene locus (Table 1). (**D**) (Top) Co-expression of EAT-4 and UNC-17 results in cytosolic mCherry. (Bottom) Cytosolic mCherry was detected in the head of three neurons: (**i**) AFDR, AFDL, and M5; and in two neurons in the tail region: (ii) PVN and DVA. (**E**) Fluorescence microscopy shows neurons with co-expression of (**E**) VGLUT/EAT-4 and VGAT/UNC-47; (**F**) VGLUT/EAT-4 and dopamine synthesis gene DAT-1; (**G**) VAChT/UNC-17 and VGAT/UNC-47; and (**H**) VAChT/UNC-17 and the serotonin synthesis gene TPH-1. All scale bars = 10 μm.

Co-transmission is a conserved feature of neurons across the animal kingdom (Granger et al., 2017; Trudeau and El Mestikawy, 2018; Vaaga et al., 2014), including *C. elegans* (Duerr et al., 2008; Gendrel et al., 2016; Pereira et al., 2015; Pocock and Hobert, 2010; Serrano-Saiz et al., 2017; Serrano-Saiz et al., 2013; Taylor et al., 2021; Wang et al., 2024), but the prevalence of co-transmission in vivo for any given organism is not well understood. To validate the utility of our tools and to map the architecture of co-transmission in *C. elegans*, we developed an intersectional genetic strategy using Flippase recombinase and FRT-flanked fluorescent reporters (Figure 6A). We focused on identifying neurons that co-transmit glutamate or acetylcholine—the two most abundant excitatory neurotransmitters in *C. elegans*—in combination with other transmitters (Figure 6B).

**Figure 6—figure supplement 1.**
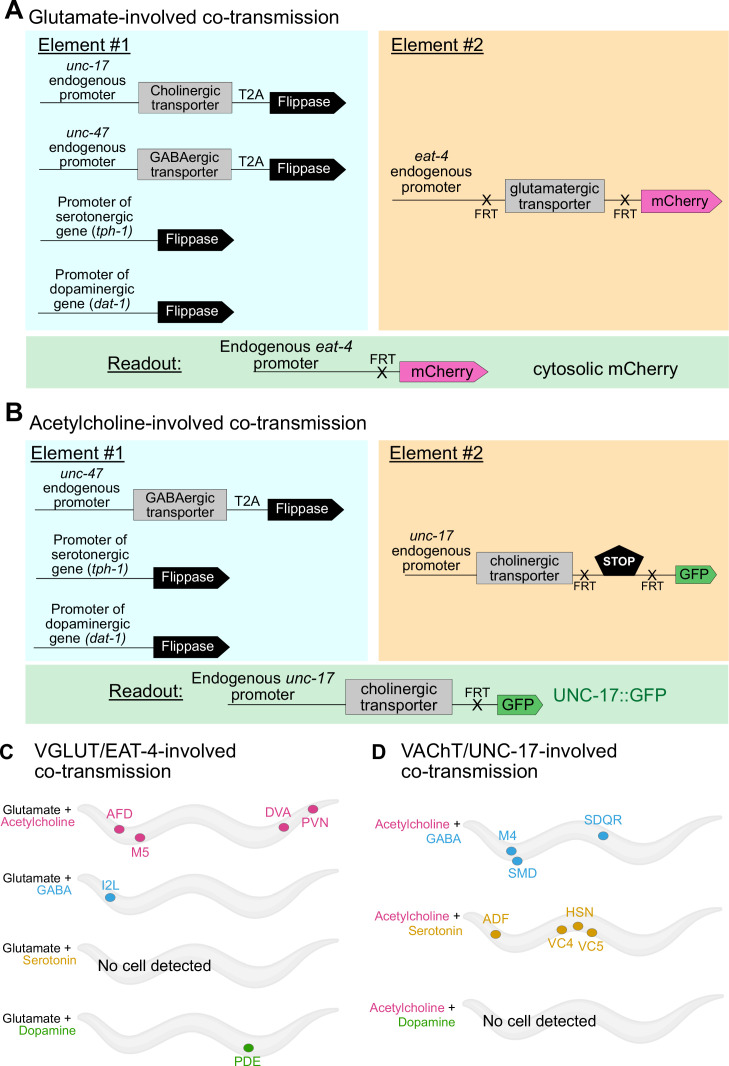
Genetic strategy that reveals potential co-transmitter neurons. (**A**) To track glutamate-involved co-transmission, we repurposed the *eat-4* conditional KO strain (*kySi76 kySi77*) (López-Cruz et al., 2019) where the *eat-4* gene coding sequence is flanked by two FRT sites and followed by cytosolic mCherry (Element #2, orange box). Crossing this line with a panel of flippase drivers (Element #1, blue box) results in activation of cytosolic mCherry only in cells where both elements were co-expressed (Readout, green box). P*dat-1*::Flippase and P*tph-1*::Flippase driver strains were made available from Muñoz-Jiménez et al., 2017. (**B**) To track acetylcholine-involved co-transmission, we repurposed the unc-17::GFP conditional KI strain (*ola503*) (Figure 3B) for which, after the *unc-17* gene coding sequence, there are FRT sites flanking the STOP codon and 3’ UT, followed by GFP (Element #2, orange box). Crossing this line with a panel of flippase drivers (Element #1, blue box) results in the protein fusion of UNC-17 with GFP in cells where both elements were co-expressed (Readout, green box). (**C, D**) Systematic mapping shows neurons with co-transmission of (**C**) glutamate in combination with acetylcholine, GABA, and dopamine. (**D**) Co-transmission of acetylcholine is found in combination with GABA and serotonin.

**Figure 6—figure supplement 2.**
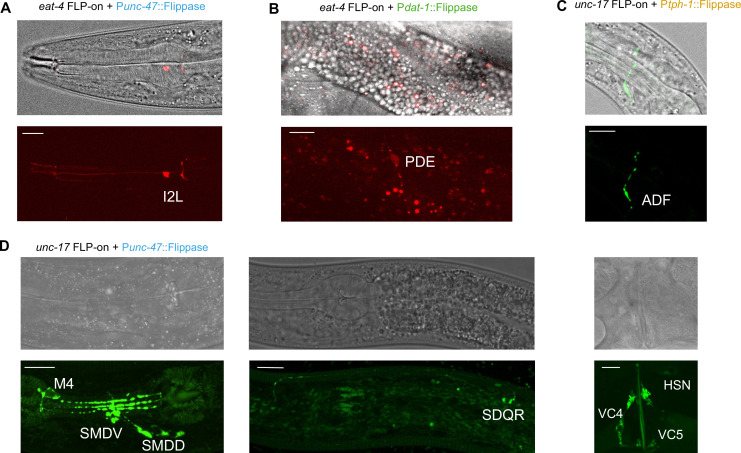
Live imaging of co-transmitter neurons identified. Live-imaging of co-expression strategy of the *eat-4* conditional knockout strain (*kySi76 kySi77*) with (**A**) endogenous P*unc-47*::UNC-47::T2A::Flippase and (**B**) P*dat-1*::Flippase driver. Live-imaging of co-expression strategy of the *unc-17* conditional GFP knock-in strain (*kySi76 kySi77*) with (**C**) P*tph-1*::Flippase driver, and with (**D**) endogenous P*unc-47*::UNC-47::T2A::Flippase. (Top) DIC images. (Bottom) Fluorescence imaging. All scale bars = 10 μm.

**Figure 6—figure supplement 3.**
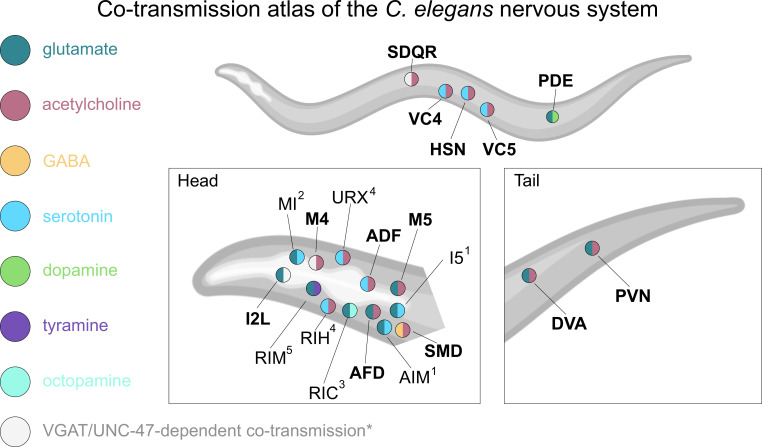
Co-transmission atlas of the *C. elegans* nervous system. Our results (bold) are consistent and extend findings from previous studies in the field (see Table 4 and numbered references below). Here, we present an atlas of co-transmission in the *C. elegans* nervous system. Co-transmitter neurons are present in the (i) head, mid-body region, and (ii) tail of the animal. *Note that I2L, M4, and SDQR express UNC-47/VGAT but do not stain positive for GABA staining (Gendrel et al., 2016) nor express genes required for GABA synthesis or uptake. ^1^Jafari et al., 2011; Serrano-Saiz et al., 2013; Wang et al., 2024. ^2^Serrano-Saiz et al., 2013; Wang et al., 2024. ^3^Reilly et al., 2022; Wang et al., 2024. ^4^Jafari et al., 2011; Wang et al., 2024. ^5^Alkema et al., 2005; Serrano-Saiz et al., 2013; Wang et al., 2024.

We reasoned that if two neurotransmitters were co-expressed in the same neuron, driving Flippase under the promoter of one transmitter would activate the conditional reporter—resulting in fluorescence—only in cells also expressing a second neurotransmitter identity (Figure 6A, B). To achieve this, we used the engineered alleles for each vesicular transporter that we developed (Figures 2B–5B—5, Table 1) and developed additional Flippase driver by modifying the locus of genes involved in the packaging of acetylcholine (*unc-17*) and GABA (*unc-47*) (Table 1). Additionally, we used available Flippase driver lines for serotonin and dopamine (Figure 6—figure supplement 1A, B, Table 1; Muñoz-Jiménez et al., 2017).

We first used a conditional *eat-4/VGLUT* reporter strain in which cytosolic mCherry is expressed upon Flippase-mediated recombination (López-Cruz et al., 2019). When Flippase was driven by the *unc-17/VAChT* promoter (cholinergic), we observed five mCherry-positive neurons in the head and tail, consistent with co-expression of *unc-17/VAChT* and *eat-4/VGLUT*. Based on cell position, neurite morphology, transcriptomic data (Taylor et al., 2021), and anatomical maps (Wang et al., 2024), we identified these neurons as AFDL, AFDR, M5, DVA, and PVN (Figure 6C, D).

Flippase expression from the GABAergic *unc-47* promoter activated *eat-4*-driven mCherry expression in a single pharyngeal neuron, identified as I2L (Figure 6E, Figure 6—figure supplement 2A). Driving Flippase from the dopaminergic *dat-1* promoter labeled the PDE neuron (Figure 6F, Figure 6—figure supplement 2B), while serotonergic *tph-1*-driven Flippase did not produce any mCherry-positive neurons. These results are summarized in Figure 6—figure supplement 1C.

We applied a similar strategy by using our Flippase-dependent *unc-17*::GFP reporter to identify candidate neurons that co-release acetylcholine with other neurotransmitters. In this context, when Flippase was driven from the GABAergic *unc-47* locus, we observed GFP expression in the M4, SDQR, and SMD neurons (Figure 6G, Figure 6—figure supplement 2D). Flippase expression from the serotonergic *tph-1* promoter revealed previously described acetylcholine/serotonin co-transmitting neurons, including ADF, HSN, and VC4/VC5 (Figure 6H, Figure 6—figure supplement 2G), consistent with prior findings (Pereira et al., 2015). All neurons identified through this dual-reporter approach are summarized in Figure 6—figure supplement 1C.

We compared our findings with previous reports and have compiled a list of potential co-transmitter neurons that are consistently identified across independent studies (Figure 6—figure supplement 3), suggesting these are likely *bona fide* co-transmitter neurons. Together, we observe that *C. elegans* has 35 neurons exhibiting molecular signatures of co-transmission (Table 3)—representing ~10% of the *C. elegans* nervous system (Figure 6—figure supplement 3 and Table 4). Strikingly, the pharyngeal nervous system—analogous to the vertebrate enteric nervous system (Albertson and Thomson, 1976)—had the highest density of co-transmitter neurons: 30% (6 of 20 neurons) displayed co-expression of multiple vesicular transporters. Across the entire nervous system, co-transmission was prevalent among sensory neurons, with 12% (10 of 83), compared to 11% of interneurons (9 of 81) and 7% of motor neurons (8 of 116) (Table 3). We also compiled a list of neurons that were suggested to be co-transmitters (RIB, AVA, and AVB) (Gendrel et al., 2016), but that are not supported by other expression studies (Taylor et al., 2021; Table 5).

**Table 3.** List of potential co-transmitter neurons by neuronal type.

Pharyngeal neurons	Sensory neurons	Interneurons	Motor neurons
I2L	ADFL	AIML	HSNL
I5	ADFR	AIMR	HSNR
M4	AFDL	RIH	SMDDL
M5	AFDR	PVNL	SMDDR
MI	URXL	PVNR	SMDVL
	URXR	RIML	SMDVR
	SDQR	RIMR	VC04
	DVA	RICL	VC05
	PDEL	RICM	
	PDER		

**Table 4.** List of potential co-transmitter neurons reported by at least more than one study.

Co-transmitter neurons	Suggested by CenGEN expression data?^*^	Consistent with previous reports	Combination suggested
**ADF**	Yes	Pereira et al., 2015, Wang et al., 2024, and this study	Serotonin + acetylcholine
**AFD**	Yes	Wang et al., 2024 and this study	Acetylcholine + glutamate
AIM	No	Jafari et al., 2011, Serrano-Saiz et al., 2013, Wang et al., 2024	Serotonin + glutamate
**DVA**	Yes	Wang et al., 2024 and this study	Acetylcholine + glutamate
**HSN**	Yes	Duerr et al., 2001, Wang et al., 2024, and this study	Serotonin + acetylcholine
**I2L**	No	Wang et al., 2024 and this study	Glutamate + UNC-47-dependent NT
I5	No	Jafari et al., 2011, Serrano-Saiz et al., 2013, Wang et al., 2024	Serotonin + glutamate
**M4**	No	Wang et al., 2024 and this study	Acetylcholine + UNC-47-dependent NT
**M5**	Yes	Wang et al., 2024 and this study	Acetylcholine + glutamate
MI	Yes	Serrano-Saiz et al., 2013, Wang et al., 2024	Serotonin + glutamate
**PDE**	No	Wang et al., 2024 and this study	Dopamine + glutamate
**PVN**	Yes	Wang et al., 2024 and this study	Acetylcholine + glutamate
RIC	Yes	Reilly et al., 2022, Wang et al., 2024	Glutamate + octopamine
RIH	Yes	Jafari et al., 2011, Wang et al., 2024	Serotonin + acetylcholine
RIM	Yes	Alkema et al., 2005, Serrano-Saiz et al., 2013, Wang et al., 2024	Glutamate + tyramine
**SDQR**	Yes	Wang et al., 2024 and this study	Acetylcholine + UNC-47-dependent NT
**SMD**	Yes	Gendrel et al., 2016 and this study	GABA + acetylcholine
URX	No	Jafari et al., 2011, Wang et al., 2024	Serotonin + acetylcholine
**VC04**	Yes	Duerr et al., 2001, Wang et al., 2024, and this study	Serotonin + acetylcholine
**VC05**	Yes	Duerr et al., 2001, Wang et al., 2024, and this study	Serotonin + acetylcholine

* Determined with threshold of 1.

**Table 5.** List of neurons inconsistent across studies to suggest co-transmission capacity.

Previosly suggested:	Co-transmitter combination suggested	Reported by:	Maximum CenGEN threshold that shows expression of each gene				
			cha-1	unc-17	unc-25	unc-47	unc-46
RIB	GABA +acetylcholine	Gendrel et al., 2016			4	4	4
AVB	GABA +acetylcholine	Gendrel et al., 2016	3	3			1
AVA	GABA +acetylcholine	Gendrel et al., 2016	2	3			

### In vivo visualization of co-transmitter synapses in the ADF chemosensory neuron

We next used our toolkit to investigate the subcellular localization of vesicular transporters in a co-transmitting neuron, ADF. The ADF neurons are a bilaterally symmetric pair of sensory neurons in *C. elegans* known to regulate food exploration, chemotaxis, and entry into the lethargic-like dauer state (Bargmann and Horvitz, 1991). While ADF has long been known to use serotonergic neurotransmission, our findings indicate that it is also capable of acetylcholine synthesis and packaging (Figure 6H, Figure 6—figure supplement 1C, and Figure 7—figure supplement 1A, B). Our findings are consistent with recent transcriptomic and reporter-based studies (Pereira et al., 2015; Taylor et al., 2021; Wang et al., 2024).

To examine the subcellular distribution of serotonin or acetylcholine vesicular transporters in ADF, we used previously developed tools to endogenously label the serotonin vesicular transporter CAT-1/VMAT (Huang et al., 2023), and the acetylcholine vesicular transporter UNC-17/VAChT with GFP (Figures 4 and 5). To label these vesicular transporters specifically in ADF, we drove Flippase and GFP1-10 expression using the AFD-specific promoter, *srh-142* promoter (Maicas et al., 2021). Both UNC-17/VAChT and CAT-1/VMAT displayed punctate fluorescence along the ADF axon, consistent with the location of presynaptic sites expected from electron microscopy-based 3D reconstructions (Figure 7—figure supplement 1C; Koonce et al., 2024; White et al., 1986).

**Figure 7.**
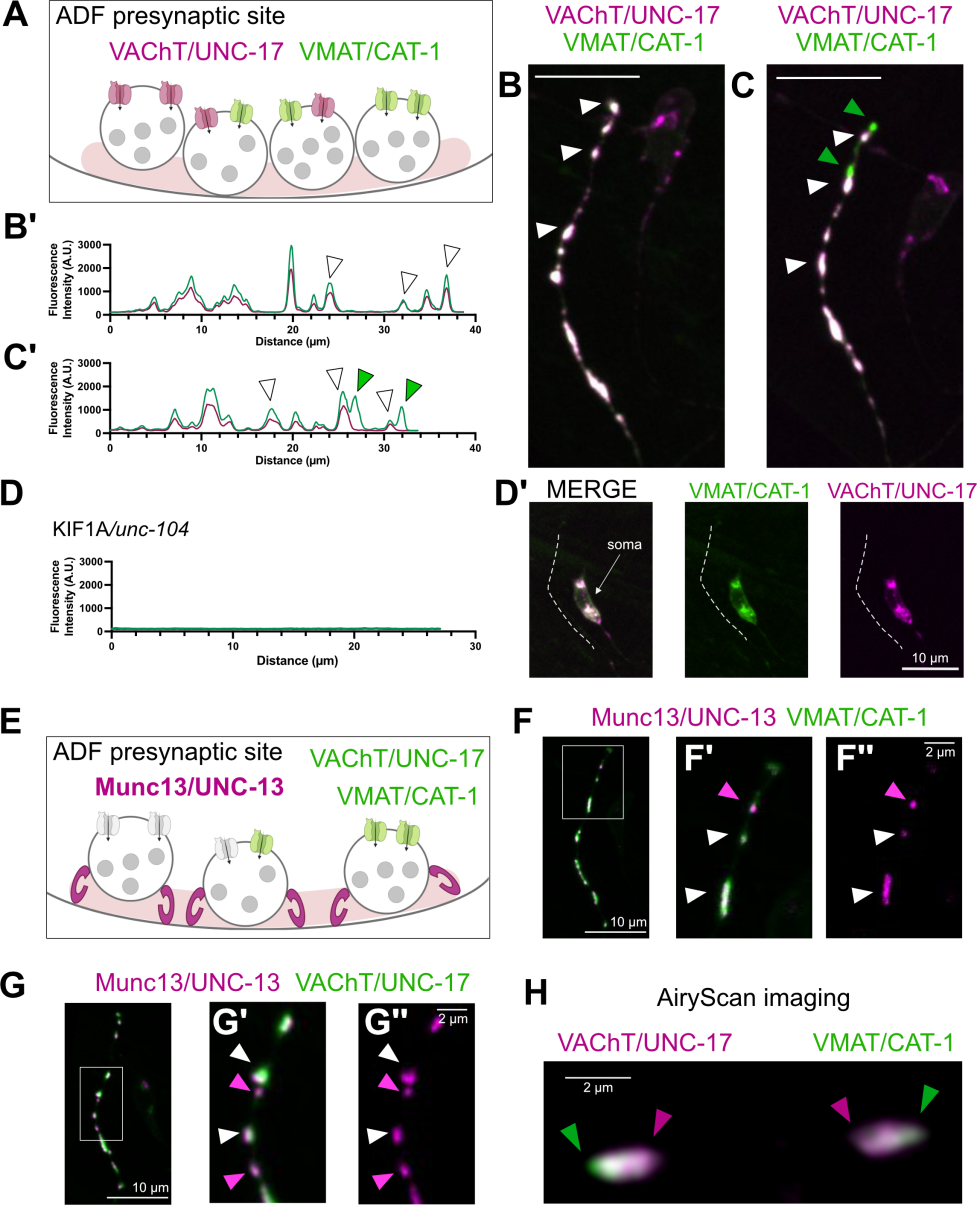
Visualizing the distribution of cholinergic and serotonergic vesicles in ADF neurons. (**A**) Dual-labeling of the endogenous acetylcholine (UNC-17, magenta) and serotonin (CAT-1, green) vesicular transporters in ADF neurons. (**B**) UNC-17::mRuby and CAT-1::GFP overlap along the ADF axon. White arrowheads denote overlap of both signals. (**B’**) UNC-17::mRuby and CAT-1::GFP intensity profile. (**C**) Example of UNC-17::mRuby and CAT-1::GFP when they partially do not overlap along the ADF axon, green arrowheads point to CAT-1-only puncta. (**C’**) UNC-17::mRuby and CAT-1::GFP intensity profile. Scale bar = 10 μm. (**D, D’**) Endogenous UNC-17::mRuby and CAT-1:::GFP are absent from the axon of *unc-104* mutant animals. Line scan of axon (**D**) and fluorescent images (**D’**). (**E**) Schematic of the dual-labeling of endogenous CAT-1::GFP or UNC-17::GFP with endogenous active zone protein UNC-13::mScarlet along the ADF axon. (**F**) Live imaging of the ADF axon reveals UNC-13::mScarlet puncta that lack CAT-1::GFP. Scale bar = 10 μm. (**F’, F’’**) Zoom-in region of E. White arrowheads show overlapping vesicular transporter tagged (green) with the active zone protein UNC-13 (magenta) in individual puncta. Magenta arrowhead points to UNC-13::mScarlet puncta that do not overlap with CAT-1::GFP. (**G**) Live imaging of the ADF axon reveals UNC-13::mScarlet puncta that lack UNC-17::GFP. Scale bar = 10 μm. (**G’, G’’**) Zoom-in region of F. White arrowheads show overlapping vesicular transporter tagged (green) with the active zone protein UNC-13 (magenta) in individual puncta. Magenta arrowhead points to UNC-13::mScarlet puncta that do not overlap with CAT-1::GFP. Scale bar = 2 μm. (**H**) AiryScan imaging of dual-labeled UNC-17::mRuby and CAT-1::GFP along the ADF axon shows localization in the same synaptic bouton but with distinct enrichment areas. Green arrowhead points to CAT-1 enrichment and magenta arrowhead points to UNC-17 enrichment. Scale bar = 2 μm. Panel A was created with BioRender.com. Panel E was created with BioRender.com.

To then visualize the spatial relationship between these two vesicular transporters, we generated a strain in which UNC-17/VAChT was tagged with mRuby and CAT-1/VMAT with GFP at their respective endogenous loci, cell-specifically in ADF neurons (Figure 7A). In vivo imaging using a spinning disk confocal microscope revealed that these transporters frequently co-localize within the same synaptic boutons (Figure 7B, B’). Interestingly, we also found instances in which the UNC-17/VAChT::mRuby and CAT-1/VMAT::GFP signals partially segregate into separate boutons along the same axon (Figure 7C, C’), suggesting that these vesicular transporters can be sorted into distinct vesicle populations. Consistent with the idea that UNC-17 and CAT-1 localizes to synaptic vesicles along the axon, we observed that UNC-17 and CAT-1 localization was disrupted in a Kif1A/*unc-104* (*e1265*) mutant animal (Hall and Hedgecock, 1991), absent in the axon and accumulated in the cell body (Figure 7D–D’).

**Figure 7—figure supplement 1.**
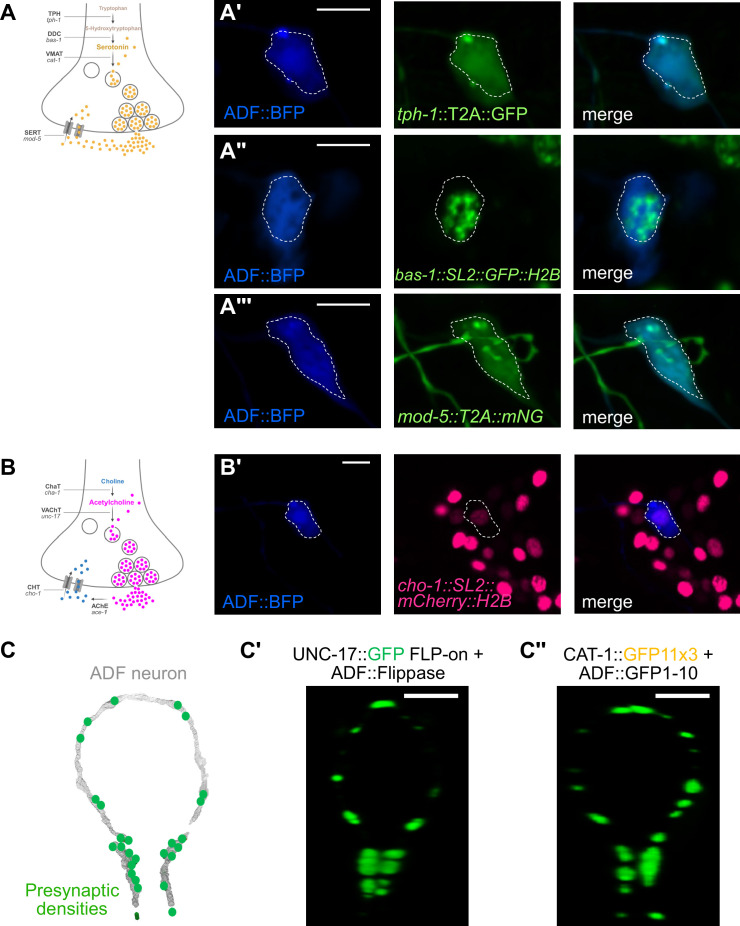
Expression of cholinergic and serotonergic genes in ADF neurons. (**A**) Schematic of genes required for serotonergic identity. Mammalian (bold) and *C. elegans* (italics) homolog genes are listed. Detectable expression of (**A’**) *tph-1,* (**A’’**) *bas-1*, and (**A’’’**) *mod-5* in ADF neuron (blue). Scale bar = 5 μm. (**B**) Schematic of genes required for cholinergic identity. Mammalian (bold) and *C. elegans* (italics) homolog genes are listed. Detectable expression of (**B’**) *cho-1* in ADF neuron (blue). Scale bar = 10 μm. (**C**) Presynaptic densities (green) on ADF axons as determined by serial electron microscopy reconstructions (White et al., 1986) image generated with NeuroSC [https://neurosc.net/]; (Koonce et al., 2024). (**C’**) 3D rendering of live fluorescence imaging of endogenous UNC-17::GFP and (**C’’**) CAT-1::GFP specifically in ADF neurons. Scale bar = 10 μm. Panel A and B was created with BioRender.com.

To test whether both transporters are present at all ADF synapses, we endogenously tagged the active zone protein UNC-13/Munc13 with mScarlet and examined its spatial relationship to UNC-17/VAChT::GFP and CAT-1/VMAT::GFP (Figure 7E). We observed active zone UNC-13::mScarlet puncta that lacked either CAT-1/VMAT::GFP (Figure 7F) or UNC-17/VAChT::GFP labeling (Figure 7G). These findings are consistent with the idea that, while these two vesicular transporters display co-localization in synaptic varicosities, they also independently localize to distinct subcellular compartments.

Synaptic varicosities in *C. elegans* can be within the diffraction limits of light microscopy, preventing differentiation of co-localizing vesicular populations. To better understand the relative distribution of these vesicular transporters, we next visualized ADF synapses with increased resolution, using AiryScan imaging, which can achieve differentiation of fluorophores up to 120 nm apart (Wu and Hammer, 2021). We observed that, even in synaptic boutons in which the vesicular transporters were observed to co-localize with traditional light microscopy methods, UNC-17/VAChT::mRuby and CAT-1/VMAT::GFP differentially segregated when imaged using AiryScan microscopy (Figure 7H).

Together these results suggest that acetylcholine and serotonin co-localize to synapses, but might be packaged into distinct vesicles with specific synaptic subcellular localization that is detectable upon super-resolution microscopy. Our findings underscore the importance of endogenous labeling in determining the specific localization of these vesicular transporters and their use with higher-resolution imaging methods, highlighting the value of the tools developed in this study to understand the cell-biological organization of synapses in vivo, particularly for neurons using more than one neurotransmitter.

## Discussion

The integration of anatomical connectivity, molecular identity, neural activity, and transmitter usage provides a powerful framework for building models of neural circuit function. The *C. elegans* community has access to a complete connectome (White et al., 1986); the cellular identity of all neurons (Sulston and Horvitz, 1977; Sulston et al., 1983); whole-brain calcium imaging (Nguyen et al., 2016; Prevedel et al., 2014; Schrödel et al., 2013); single-cell transcriptomic profiles (Taylor et al., 2021); and a full neurotransmitter identity map for all neurons (Wang et al., 2024). These datasets have inspired models describing how specific circuits may give rise to behavior. However, validating these models in vivo requires tools that can precisely manipulate the molecular components of individual synaptic connections (Dag et al., 2023; Hawk et al., 2018; Kumar et al., 2024). SynaptoTagMe provides that missing capability (Table 1) for the neurotransmitter systems that cover approximately 90% of the *C. elegans* nervous system (GABA, glutamate, acetylcholine, and the monoamines). By enabling cell-specific labeling and conditional knockout of vesicular transporters, we can now directly test the contribution of individual neurotransmitters within defined circuits and link those changes to behavioral outcomes. Moreover, due to the evolutionary conservation of vesicular transporters, the in vivo validation of tagging strategies will help identify suitable labeling strategies for other organisms, providing a path toward comparative and cross-species studies of synaptic dynamics based on neurotransmitter identity.

Co-transmission is a conserved feature of neural systems across the animal kingdom (Granger et al., 2017; Lacin et al., 2019; Vaaga et al., 2014), but its preponderance in vivo, its regulation and its functional significance is still an area of active research. Using in vivo reporters subject to endogenous regulation, and contrasting our results with previous studies, we determine that more than 10% of *C. elegans* neurons have co-transmission potential (Figure 6—figure supplement 3; Tables 2 and 3). Our in vivo characterization of co-transmitting neurons confirms and expands findings reported for the latest neurotransmitter atlas of *C. elegans* (Wang et al., 2024) and yield three key insights. First, co-transmission occurs throughout the nervous system of *C. elegans*, including both the pharyngeal (enteric-like) and more central nervous systems, like the nerve ring and nerve cords (Table 3). Second, neurons can co-transmit multiple neurotransmitters in specific combinations that are conserved from nematodes to mammals (Figure 6, Figure 6—figure supplement 3; Granger et al., 2017; Lacin et al., 2019; Trudeau and El Mestikawy, 2018; Vaaga et al., 2014; Wang et al., 2024). Importantly, the same neurons consistently exhibit co-transmission of the same neurotransmitter identities across individual animals, consistent with co-transmitter identity mapping to neuronal identity (Figure 6D–H). Third, co-transmission is part of every layer of a circuit, from sensory neurons to interneurons and motor neurons (Table 3). This is especially interesting in light of recent studies showing that co-transmission in sensory and motor circuits can be modulated by environmental cues such as stress (Bertuzzi et al., 2018; Li et al., 2024; Pocock and Hobert, 2010) or light–dark cycles (Chen et al., 2023; Maddaloni et al., 2024). With the tools developed here—based on endogenously tagged vesicular transporters—it is now possible to monitor the dynamic expression and subcellular distribution of specific vesicle populations in vivo (Figure 7) and what molecular mechanisms drive those changes.

Our conclusions are supported by independent and convergent lines of evidence, and the toolkit developed here enables direct empirical interrogation of both the existence and functional relevance of co-transmission in vivo. While expression of vesicular transporters represents one line of evidence for co-transmission potential, it does not by itself establish functional co-release, which additionally depends on neurotransmitter biogenesis, release competence, and activity-dependent regulatory mechanisms. SynaptoTagMe now makes it possible to systematically test these requirements.

We note that the current characterization of co-transmitting neurons might be an underestimate of the total number of neurons which use co-transmission. For example, it has been proposed that additional neurotransmitters, like betaine, may function in the *C. elegans* nervous system (Wang et al., 2024). Accounting for neurons that express proteins capable of synthesizing or packaging betaine, the proportion of potential co-transmitter neurons may exceed 20% of the whole nervous system of *C. elegans*. Our characterization of co-transmission focused on the *C. elegans* adult hermaphrodite, and co-transmitting neuron identities could be developmentally regulated, or modulated based on prior experience. Consistent with this, it has been observed that the identity of co-transmitting neurons is different between males and hermaphrodites (Serrano-Saiz et al., 2017), underscoring the importance of future examination of the plasticity and developmental regulation (Pereira et al., 2019; Pereira et al., 2015) of co-transmitting capacity for individual neurons.

Expression of a vesicular transporter, while consistent with co-transmitting capacity, is not conclusive for the existence of co-transmission for that neuron. For example, we identified co-expression of the GABA and glutamate vesicular transporters in the pharyngeal neurons I2 (Figure 6—figure supplement 2A). Notably, I2 does not express the GABA synthesis enzyme, *unc-25*/GAD (McIntire et al., 1993), or the GABA re-uptake transporter, *snf-11* (Mullen et al., 2006). Thus, it is unlikely that it produces GABA or uptakes it from the extracellular space. VGAT/UNC-47 has also been reported to transport neurotransmitters such as glycine (Aubrey et al., 2007) and beta-alanine (Juge et al., 2013), raising the possibility that I2 could co-transmit glutamate with an unconventional neurotransmitter. Additionally, it is important to mention that the identification of *eat-4*-positive neurons through the replacement of the *eat-4* coding sequence and introns (Figure 6—figure supplement 1A) could result in the elimination of regulatory sequences. Thus, we conceptualize the list of co-transmitting neurons as a hypothesis-generating framework to be further examined with the tools developed in this study.

Our observations of the identity of co-transmitting neurons, and the specific combinations represented in the neurons, suggest that there may be transmitter-specific rules of synaptic biology important for circuit function (Silm et al., 2019; Trudeau and El Mestikawy, 2018). This is consistent with findings in vertebrates, in which specific neurotransmitter combinations and their distributions could underpin specific features of circuit function. For example, in starburst amacrine cells in the mammalian retina, acetylcholine and GABA (O’Malley et al., 1992) are packaged into distinct vesicle pools that exhibit different calcium sensitivities for release (Lee et al., 2010). The distribution of specific vesicular populations and their release probabilities might constitute an architecture that helps encode the sensory signals processed by starburst amacrine cells. We similarly hypothesize that the specific distribution of co-transmitting synapses across the *C. elegans* connectome, and the identities of the neurotransmitters used, might help encode features important for circuit function and animal behavior. By allowing longitudinal, cell-specific monitoring of the expression, regulation, and subsynaptic distribution of distinct vesicle populations in living animals, this toolkit provides a platform for probing when, where, and how co-transmission is deployed, and for defining the molecular and circuit-level mechanisms that govern its use under different physiological and environmental conditions.

## Methods

### Strains

Worms were maintained at 20°C using standard techniques (Brenner, 1973). Strains were maintained on Nematode Growth Medium (NGM) plates seeded with *E. coli* (OP-50). The wild type (WT) is N2, and only hermaphrodite worms were used for this study. A complete list of strains appears below. Strains that were developed in this study as part of the SynaptoTagMe toolkit and appear in Table 1 can be requested through CGC. The necessary sequencing information can be found here.

### Generation of new alleles

For the strains engineered by Sunybiotech, as described below, strain design was performed in the Colón-Ramos lab by Andrea Cuentas-Condori.

Sunybiotech used CRISPR/Cas9 to insert GFP FLP-on cassettes (Schwartz and Jorgensen, 2016) at either the N-termini of the *unc-17* locus (*syb7251*) or C-termini end of the *unc-17 (ola503)*, or *eat-4 (syb8568)* locus, according to sequence design. mRuby3 FLP-on cassettes were inserted similarly at the C-termini end of *eat-4* (*syb9193*) and *unc-17* (*syb7882*) locus. To visualize the fluorescent signal tagged to the protein of interest, Sunybiotech generated single-copy MosCI strains.

Sunybiotech used CRISPR/Cas9 to add full-length GFP (*syb6990*), full-length mKate (*syb7358*), or split GFP (one (*syb7313*) or three (*syb7849*) copies of GFP11) to the *unc-47* locus at the +893 bp position. To visualize the reconstituted GFP signal in RIB neurons, complementary GFP1-10 was driven with the P*sto-3b* promoter; and to visualize the GFP-reconstituted signal in DD neurons, GFP1-10 was driven with the P*flp-13* promoter.

Sunybiotech used CRISPR/Cas9 to introduce an FRT site before the +1 bp in the *unc-25* gene locus (*syb5949*). In a second round of CRISPR editing, they introduced *let-858* 3’ UTR followed by a second FRT and nuclear mCherry (*syb6275*).

*cat-1* (*ky1101 ky1118*) cell-specific knockout strain was created using CRISPR/Cas9 to introduce an FRT site immediately before the ATG in the *cat-1* gene locus (*ky1101*). A second round of CRISPR editing in that strain introduced the *let-858* 3’ UTR followed by a second FRT and the mCherry coding region immediately after the stop codon of *cat-1* to generate *ky1118*.

Sunybiotech used CRISPR/Cas9 to introduce a T2A::Flippase sequence before the STOP codon of *unc-47(syb8125)* and *unc-17(syb8059)*. All strains generated using CRISPR/Cas9 were outcrossed twice before use.

### Molecular biology

Plasmids were constructed using Gibson cloning. First, Snapgene (Version 7.0.3) software was used to design primers targeting the desired DNA vector backbone and DNA insert. The vector backbone and DNA insert were PCR linearized and amplified using ‘CloneAmp HiFi PCR Premix’. To assemble the desired plasmid, the purified vector backbone DNA and insert DNA were combined and incubated in solution with ‘2x Gibson Assembly Enzyme Premix’. Following incubation, the reaction mixture was used to transform Stellar Competent Cells, which were subsequently plated and grown overnight on LB-Amp plates. All plasmids were verified with Sanger sequencing.

### Protein alignment and structure visualization

For each gene under study, NCBI BLAST was used to generate a protein sequence alignment of the *C. elegans* gene with the closest orthologs from the other model organisms *M. musculus*, *D. rerio*, *D. melanogaster*, and *H. sapiens*. Protein structure models for the *C. elegans* genes were downloaded from the AlphaFold database (Jumper et al., 2021) and predicted models for the CRISPR-Cas9 modified genes including fluorophores were generated by the Alphafold3 online server (Abramson et al., 2024). Visualization and image generation of protein structures was done using the ChimeraX software (Pettersen et al., 2021). To color the structures by sequence conservation, the alignments per gene were overlaid onto the structures with ChimeraX and colored by resulting sequence conservation *Z*-scores as calculated by the AL2CO algorithm (Pei and Grishin, 2001) within the software.

### Microscopy

Larval or young adult animals were immobilized on 2–10% agarose pads with 10 mM levamisole. A Nikon Ti2 microscope equipped with a CSU-W1 spinning disk head, ORCA-Fusion BT SCMOS camera, high-speed piezo stage motor, 60X/1.40 Apo Lambda oil objective lens was used for live imaging. Z-stack images were collected (0.3–0.5 μm/step), spanning the focal depth of the nerve cord and nerve ring synapses. A Zeiss LSM880 microscope equipped with an AiryScan detector and 63X NA 1.4 oil objective was used for AiryScan imaging. FIJI (Schindelin et al., 2012) or NIS Elements AR analysis software (version 6.10.01) were used to create maximum intensity projections and 2D renderings.

### Thrashing assay

*C. elegans* were raised at 20°C under standard laboratory conditions on agar plates seeded with a lawn of *E. coli* (OP50). Worms were synchronously grown to L4-stage and placed in individual wells of a Corning PYREX Spot Plate (Catalog #722085) containing 1000 μl of M9 buffer, ensuring the buffer remained within the well’s borders. After a 30-s acclimation period to M9, thrashes were manually counted for 1 min. A single thrash was defined as a change in the direction of the worm’s midbody bending, counting each time the worm’s body flexed to one side. Following each trial, the worm was removed using a pipette and disposed of, and the M9 buffer was absorbed and discarded. The well was then cleaned with 70% ethanol and wiped dry. To avoid bias, the counter was blinded to each genotype. Each worm was tested only once, with assays conducted on 10 worms per genotype per day, and repeated over 2–3 days to account for potential day-to-day environmental variations.

### Chemotaxis assay

Worms were maintained at 20°C for at least two generations on NGM seeded with OP50 *Escherichia coli* bacteria. The concentration of our attractant (NaCl) is approximately 50 mM in NGM plates. ‘Training’ plates were produced using NGM with the further addition of 50 mM NaCl to a total concentration of 100 mM, then also seeded with OP50.

Chemotaxis assay was modified from standard procedure (Ward, 1973). All assays were performed on 50 mm diameter plates. Unseeded NGM plates were marked at the center and one point 12.5 mm away from the center. A ~60–85 mM gradient of NaCl was created between the center and outer point by adding 5 M NaCl at the outer point as drops of 4 μl (20–24 hr before the assay), 4 μl (5 hr before), and 1.6 μl (2 hr before); a sham gradient was created using only water. Gradient prediction was determined as previously described (Crank, 1956; Pierce-Shimomura et al., 1999); briefly, for every point some distance *r* in cm from the salt peak, the concentration *C* in mM at any point in time was calculated as:

(1) $$\displaystyle  C\left (t_{i},r\right)=C_{o}+\sum _{i=1}^{n}c\left (t_{i},r\right),$$

where *C_o_* is the initial concentration of NaCl in the agar (50 mM), *n* is the drop number, and *t_i_* is the time in seconds since the drop had been applied; the contribution from each drop, in turn, was calculated as:

(2) $$\displaystyle c\left (t_{i},r\right)=10^{6}\frac{N_{i}}{\sqrt{4\pi dD\left (t_{i}\right)}}e^{- \left (\frac{r^{2}}{4D\left (t_{i}\right)}\right)},$$

where *N_i_* is the moles of NaCl added per drop; *d* is the depth (cm) of the agar; and *D* is 1.590 × 10^–5^ cm^2^/s, the diffusion coefficient for 5 M NaCl through an aqueous medium (Robinson and Stokes, 1959). The resulting gradients were validated by electrical conductivity measurements using an Oakton CON 6+ Handheld Conductivity Meter with a custom conductivity probe with 1 mm insertion depth (Micro-electrodes, Inc, Bedford, NH). The conductivity readings from 50 and 100 mM NaCl NGM plates were used for calibration at specific room temperatures.

The day before experiments, L4 animals were transferred to a seeded NGM plate to synchronize worms by developmental stage. At 5 hr before each assay, worms were transferred to a training plate using standard NGM plate recipe adjusted to 100 mM NaCl. After training, eight worms were picked, with preference to those on the bacterial lawn, washed sequentially in two 100 µl drops of liquid NGM buffer (25 mM potassium phosphate pH 6, 1 mM CaCl_2_, 1 mM MgSO_4_, 50 mM NaCl) to remove adherent OP50, and transferred to a single 2 μl drop of NGM buffer at the center of the assay plate with prepared NaCl gradient as described. Data collection began when the water drop was fully absorbed into the assay plate and the first worm began to migrate from its starting point. Six assay plates were imaged for each strain across two separate days, yielding a total of 48 worms imaged per strain.

Images of chemotaxis behavior were acquired at 3.75 fps for 7 min using a Basler acA2440-35mm monochromatic sensor with an infrared filter on a commercially available WormLab imaging system and computer running WormLab 2023.1.1 software (MBF Bioscience LLC, Williston, VT USA). Individual worm position data was obtained by constructing tracks in WormLab software, then analyzed using custom scripts in R 4.4.1 (can be accessed through GitHub; https://github.com/colonramoslab/Cuentas-Condori-et-al.-2025-Toolkit-; copy archived at Thomas, 2025). The assay outcome was defined as the mean distance from the peak of the salt gradient for each worm, averaged over every available frame in the last minute of the assay. When a worm track was interrupted, e.g. by a worm exiting the camera field of view or by two worms intersecting, the last available position for the worm was repeated until the worm was re-detected.

### Roaming assay

Roaming assay plates were prepared 3–5 days prior to the experiment by seeding NGM agar plates with *E. coli* (OP50) culture no older than 2 days. Plates were seeded using a sterile glass rod to spread the bacteria evenly across each plate. Plates were left to dry completely between 3 and 5 days at room temperature to ensure the bacteria layer was fully dry, thereby allowing for visible worm tracks during the assay. *C. elegans* were raised at 20°C under standard laboratory conditions on agar plates seeded with a lawn of *E. coli* (OP50). On the day before the assay, worms synchronously grown to L4-stage were transferred to regular seeded plates and stored at 20°C. After approximately 10 hr, worms were transferred to individual assay plates and incubated at 20°C for 16 hr. After this time, worms were removed and a grid overlay (3 mm × 3 mm squares) covering the assay plate was used to count the number of squares the worms had traversed during the incubation period. The number of squares crossed provided a quantifiable measure of roaming activity. To avoid bias, the counter was blinded to each genotype. Each worm was tested only once, with assays conducted on 10 worms per genotype per day and repeated over 2–3 days to account for potential day-to-day environmental variations.

### Aversion behavior assay

Aversion behavior assay were performed as previously described (Feng et al., 2025). Animals were fed on *E. coli* BW25113 for at least three generations before the behavioral assay. 12.5 μl of overnight BW25113 cultures were seeded onto standard NGM agar plates, grown at 37°C in an incubator for 24 hr and then left at room temperature for another 24 hr. 15–20 animals at L4 stages from each genotype were transferred onto behavioral assay plates and recorded at 21°C for 20 hr, at a recording rate of 1 frame per minute. Biological replicates across two different days were conducted. Videos were cropped and analyzed using standard MatLab codes (Marquina-Solis et al., 2024). Aversion ratio was defined by the number of worms outside the bacterial lawn over the total number of worms on assay plates.

### Statistical analysis

We used the Shapiro–Wilk test to determine sample distribution. For comparisons between two normally distributed groups, Student’s *t*-test was used and p < 0.05 was considered significant. ANOVA was used to compare between three or more normally distributed groups followed by Dunnett’s multiple-comparison test. If the samples were not normally distributed, we used a Mann–Whitney test to compare two groups and a Kruskal–Wallis test to compare three or more groups. Specific post hoc statistical tests are listed in the figure legend of each experiment. Prism 10.4.2 was used to graph the data and for all statistical analysis.

### List of strains

**Table:** 

Strain name	Genotype
PHX8568	eat-4::gfp FLP-on(syb8568) III
DCR9575	eat-4::gfp FLP-on(syb8568) III 2X outcrossed
MT6308	eat-4(ky5) III
DCR9690	Pflp-6::FLP(sybIs9606) II
DCR9814	Pflp-6::FLP(sybIs9606) II 2X outcrossed
DCR9872	eat-4::gfp FLP-on(syb8568) III; Peft-3::FLP (sybIs9614) II
DCR9816	eat-4::gfp FLP-on(syb8568) III; Pflp-6::FLP (sybIs9606) II
DCR9681	eat-4::mRuby FLP-on(syb9193) III 2X outcrossed
DCR9963	GFP::RAB-3 FLP-on(ox699) III; Pgcy-5::FLP (sybIs8828)
DCR9965	GFP::RAB-3 FLP-on(ox699) III; olaEx5706[Pgcy-7::Flippase::T2A::BFP; Punc-122::RFP]
	
DCR9210	unc-47::gfp (syb6990) III 2X outcrossed
DCR9453	unc-47::mKate2 (syb7358) III 2X outcrossed
CB307	unc-47(e307) III
DCR9269	unc-47::gfp (syb6990) III; olaEx5490[Psto-3b::BFP; Punc-122::RFP]
PHX7313	unc-47::gfp11 (syb7313) III
DCR9280	unc-47::gfp11 (syb7313) III 1X outcrossed
DCR9738	unc-47::gfp11 (syb7313) III; olaEx5686[Pflp-13::GFP1-10; Pmyo-2::mCherry]
PHX7849	unc-47::gfp11x3 (syb7849) III
DCR9739	unc-47::gfp11x3 (syb7849) III 2X outcrossed
DCR9740	unc-47::gfp11x3 (syb7849) III; olaEx5686[Pflp-13::GFP1-10; Pmyo-2::mCherry]
DCR9741	unc-47::gfp11x3 (syb7849) III; olaEx5687[Psto-3b::GFP1-10; Punc-122::RFP]
CF4587	muIs253 [Peft-3::GFP1-10::unc-54 3’UTR +Cbr-unc-119(+)] II; unc-119(ed3) III
DCR9838	muIs253 [Peft-3::GFP1-10::unc-54 3’UTR +Cbr-unc-119(+)] II; unc-119(ed3) III unc-47::gfp11x3 (syb7849) III recombinant
PHX6275	unc-25 (syb5949 syb6275) III
DCR9898	unc-25 (syb5949 syb6275) III; bqSi506[Prgef-1::Flippase D5+unc-119(+)] IV
	
CB933	unc-17(e245) IV
DCR9891	unc-17(e245) IV; olaEx5703[Punc-17::UNC-17cDNA]
DCR9074	unc-17(e245) IV; olaEx5400[Punc-17::GFP::UNC-17 (N-terminal tag)]
DCR9892	unc-17(e245) IV; olaEx5704[Punc-17::UNC-17::GFP (between TM6-7)]
OH15568	unc-17::mKate2 (ot907) IV
DCR9011	unc-17::GFP FLP-on(ola503) IV
DCR9211	unc-17::GFP FLP-on(ola503) IV 1X outcrossed
PHX7251	GFP FLP-on::unc-17 (syb7251) IV
DCR9265	GFP FLP-on::unc-17 (syb7251) IV 1X outcrossed
DCR9720	Peft-3::FLP (sybIs9614) II
DCR9733	Peft-3::FLP (sybIs9614) II 2X outcrossed
DCR9742	unc-17::GFP FLP-on(ola503) IV; Peft-3::FLP (sybIs9614) II
DCR9374	unc-17::mRuby FLP-on(syb7882) IV 2X outcrossed
DCR9989	unc-17::mRuby FLP-on(syb7882) IV; Peft-3::FLP (sybIs9614) II
	
PHX7239	cat-1::gfp11x3(syb7239) X
DCR9370	cat-1::gfp11x3(syb7239) X 2X outcrossed
DCR9338	cat-1::gfp11x3(syb7239) X; olaEx5516[Psrh-142::GFP1-10; Psrh-142::BFP; Punc-122::RFP]
RB681	cat-1(ok411)
DCR9837	muIs253 [Peft-3::GFP1-10::unc-54 3’UTR +Cbr-unc-119(+)] II; unc-119(ed3) III cat-1::GFP11x3(syb7239) X
DCR9414	cat-1(ky1101 ky1118) X 2X outcrossed
DCR9736	Psrh-142::FLP (syb9159) II 2X outcrossed
DCR9912	Psrh-142::FLP (syb9159) II; cat-1(ky1101 ky1118) X
DCR9342	unc-17::GFP FLP-on(ola503) IV; unc-13::mScarlet FLP-on (wy1322) I; vlcSi1[unc-119(+); Psrh-142::Flippase] III
DCR9574	unc-17::mRuby FLP-on(syb7882) IV; cat-1::gfp11x3(syb7239) X; olaIs153 [Psrh-142::GFP1-10; Psrh-142::FLP; Punc-122::RFP]
DCR9588	unc-104(e1265) II; unc-17::mRuby FLP-on(syb7882) IV; cat-1::gfp11x3(syb7239) X; olaEx5548[Psrh-142::Flippase; Psrh142::GFP1-10; Punc-122::RFP]
DCR9448	cat-1::gfp11x3(syb7239) X; unc-13::mScarlet FLP-on (wy1322) I; olaEx5550[Psrh-142::Flippase; Psrh142::GFP1-10; Punc-122::RFP]
DCR9583	bas-1(syb5923[bas-1::SL2::GFP::H2B]) III; olaex5513 [Psrh-142::BFP; Punc-122::RFP]
DCR9590	mod-5(vlc47[mod-5::T2A::mNeonGreen]); olaex5513 [Psrh-142::BFP; Punc-122::RFP]
	
DCR9577	eat-4(kySi76 kySi77) III [eat-4 cell-specific KO]; unc-17(syb8059) IV [Flippase expression in unc-17 locus]
DCR9584	eat-4(kySi76 kySi77) III [eat-4 cell-specific KO]; unc-47(syb8125) III [Flippase expression in unc-47 locus]
DCR9371	eat-4(kySi76 kySi77) III [eat-4 cell-specific KO]; bqSi614 IV [Pdat-1::Flippase]
DCR9334	eat-4(kySi76 kySi77) III [eat-4 cell-specific KO]; bqSi488 IV [Ptph-1::Flippase]
DCR9576	unc-17::GFP FLP-on(ola503) IV [unc-17 cell-specific GFP knock-in]; unc-47(syb8125) III [Flippase expression in unc-47 locus]
DCR9344	unc-17::GFP FLP-on(ola503) IV [unc-17 cell-specific GFP knock-in]; bqSi488 IV [Ptph-1::Flippase] recombinant
DCR9897	unc-17::GFP FLP-on(ola503) IV [unc-17 cell-specific GFP knock-in]; bqSi614 IV [Pdat-1::Flippase]

### List of plasmids

**Table:** 

Plasmid name	Genotype
DACR218	P*unc-122::RFP*
DACR704	P*myo-2*::mCherry
DACR4016	P*sto-3b::BFP*
DACR4027	P*unc-17*::UNC-17cDNA
DACR4033	P*unc-17*::UNC-17cDNA::GFP in TM6-7
DACR4038	P*unc-17*::GFP::UNC-17cDNA
DACR4064	P*sto-3b::GFP1-10*
DACR4078	*Psrh-142::*BFP
DACR4083	*Psrh-142::*Flippase
DCR4092	P*srh-142*::GFP1-10
pSH87	P*flp-13::*GFP1-10

## Data Availability

The source data file contains all numerical data used to generate Figure 2C, Figure 2 - Supplement 1, Figure 3E, Figure 3 - Supplement 1, Figure 3 - Supplement 2, Figure 4C, Figure 4 - Supplement 1, Figure 4 - Supplement 2, Figure 5D, and Figure 5E. All raw datasets used for three-dimensional electron microscopy reconstructions were previously generated and are available through WormAtlas (https://wormatlas.org/MoW_built0.92/MoW.html; White et al., 1986). Electron microscopy reconstructions of *C. elegans* are also accessible via the NeuroSC platform (https://neurosc.net/). The following previously published dataset was used: TaylorSR SantpereG WeinrebA BarrettA ReillyMB XuC VarolE OikonomouP GlenwinkelL McWhirterR PoffA BasavarajuM RafiI YeminiE CookSJ AbramsA VidalB CrosC TavazoieS SestanN HammarlundM HobertO MillerDM 2019Molecular topography of an entire nervous systemNCBI Gene Expression OmnibusGSE13604910.1016/j.cell.2021.06.023PMC871013034237253

## Additional files

MDAR checklist

## References

[bib1] Abramson J, Adler J, Dunger J, Evans R, Green T, Pritzel A, Ronneberger O, Willmore L, Ballard AJ, Bambrick J, Bodenstein SW, Evans DA, Hung C-C, O’Neill M, Reiman D, Tunyasuvunakool K, Wu Z, Žemgulytė A, Arvaniti E, Beattie C, Bertolli O, Bridgland A, Cherepanov A, Congreve M, Cowen-Rivers AI, Cowie A, Figurnov M, Fuchs FB, Gladman H, Jain R, Khan YA, Low CMR, Perlin K, Potapenko A, Savy P, Singh S, Stecula A, Thillaisundaram A, Tong C, Yakneen S, Zhong ED, Zielinski M, Žídek A, Bapst V, Kohli P, Jaderberg M, Hassabis D, Jumper JM (2024). Accurate structure prediction of biomolecular interactions with AlphaFold 3. Nature.

[bib2] Ahier A, Jarriault S (2014). Simultaneous expression of multiple proteins under a single promoter in *Caenorhabditis elegans* via a versatile 2A-based toolkit. Genetics.

[bib3] Albertson DG, Thomson JN (1976). The pharynx of *Caenorhabditis elegans*. Philosophical Transactions of the Royal Society of London. Series B, Biological Sciences.

[bib4] Alfonso A, Grundahl K, Duerr JS, Han HP, Rand JB (1993). The *Caenorhabditis elegans* unc-17 gene: a putative vesicular acetylcholine transporter. Science.

[bib5] Alkema MJ, Hunter-Ensor M, Ringstad N, Horvitz HR (2005). Tyramine functions independently of octopamine in the *Caenorhabditis elegans* nervous system. Neuron.

[bib6] Aubrey KR, Rossi FM, Ruivo R, Alboni S, Bellenchi GC, Le Goff A, Gasnier B, Supplisson S (2007). The transporters GlyT2 and VIAAT cooperate to determine the vesicular glycinergic phenotype. The Journal of Neuroscience.

[bib7] Bajar BT, Wang ES, Lam AJ, Kim BB, Jacobs CL, Howe ES, Davidson MW, Lin MZ, Chu J (2016). Improving brightness and photostability of green and red fluorescent proteins for live cell imaging and FRET reporting. Scientific Reports.

[bib8] Bargmann CI, Horvitz HR (1991). Chemosensory neurons with overlapping functions direct chemotaxis to multiple chemicals in *C. elegans*. Neuron.

[bib9] Bellocchio EE, Hu H, Pohorille A, Chan J, Pickel VM, Edwards RH (1998). The localization of the brain-specific inorganic phosphate transporter suggests a specific presynaptic role in glutamatergic transmission. The Journal of Neuroscience.

[bib10] Bertuzzi M, Chang W, Ampatzis K (2018). Adult spinal motoneurons change their neurotransmitter phenotype to control locomotion. PNAS.

[bib11] Brenner S (1973). The genetics of *Caenorhabidits elegans*. Genetics.

[bib12] Chaudhry FA, Reimer RJ, Bellocchio EE, Danbolt NC, Osen KK, Edwards RH, Storm-Mathisen J (1998). The vesicular GABA transporter, VGAT, localizes to synaptic vesicles in sets of glycinergic as well as GABAergic neurons. The Journal of Neuroscience.

[bib13] Chen N, Zhang Y, Rivera-Rodriguez EJ, Yu AD, Hobin M, Rosbash M, Griffith LC (2023). Widespread posttranscriptional regulation of cotransmission. Science Advances.

[bib14] Crank J (1956). The Mathematics of Diffusion.

[bib15] Crawford DC, Kavalali ET (2015). Molecular underpinnings of synaptic vesicle pool heterogeneity. Traffic.

[bib16] Dag U, Nwabudike I, Kang D, Gomes MA, Kim J, Atanas AA, Bueno E, Estrem C, Pugliese S, Wang Z, Towlson E, Flavell SW (2023). Dissecting the functional organization of the *C. elegans* serotonergic system at whole-brain scale. Cell.

[bib17] Duerr JS, Frisby DL, Gaskin J, Duke A, Asermely K, Huddleston D, Eiden LE, Rand JB (1999). The cat-1 gene of *Caenorhabditis elegans* encodes a vesicular monoamine transporter required for specific monoamine-dependent behaviors. The Journal of Neuroscience.

[bib18] Duerr JS, Gaskin J, Rand JB (2001). Identified neurons in *C. elegans* coexpress vesicular transporters for acetylcholine and monoamines. American Journal of Physiology. Cell Physiology.

[bib19] Duerr JS, Han HP, Fields SD, Rand JB (2008). Identification of major classes of cholinergic neurons in the nematode *Caenorhabditis elegans*. The Journal of Comparative Neurology.

[bib20] Edwards RH (2007). The neurotransmitter cycle and quantal size. Neuron.

[bib21] Eiden LE (1998). The cholinergic gene locus. Journal of Neurochemistry.

[bib22] Erickson JD, Eiden LE, Hoffman BJ (1992). Expression cloning of a reserpine-sensitive vesicular monoamine transporter. PNAS.

[bib23] Fei H, Grygoruk A, Brooks ES, Chen A, Krantz DE (2008). Trafficking of vesicular neurotransmitter transporters. Traffic.

[bib24] Feng L, Marquina-Solis J, Yue L, Harnagel A, Greenfeld Y, Bargmann CI (2025). Context-dependent serotonin signaling links dietary quality to foraging decisions. Nature Communications.

[bib25] Flavell SW, Pokala N, Macosko EZ, Albrecht DR, Larsch J, Bargmann CI (2013). Serotonin and the neuropeptide PDF initiate and extend opposing behavioral states in *C. elegans*. Cell.

[bib26] Gendrel M, Atlas EG, Hobert O (2016). A cellular and regulatory map of the GABAergic nervous system of *C. elegans*. eLife.

[bib27] Gouwens NW, Sorensen SA, Baftizadeh F, Budzillo A, Lee BR, Jarsky T, Alfiler L, Baker K, Barkan E, Berry K, Bertagnolli D, Bickley K, Bomben J, Braun T, Brouner K, Casper T, Crichton K, Daigle TL, Dalley R, de Frates RA, Dee N, Desta T, Lee SD, Dotson N, Egdorf T, Ellingwood L, Enstrom R, Esposito L, Farrell C, Feng D, Fong O, Gala R, Gamlin C, Gary A, Glandon A, Goldy J, Gorham M, Graybuck L, Gu H, Hadley K, Hawrylycz MJ, Henry AM, Hill D, Hupp M, Kebede S, Kim TK, Kim L, Kroll M, Lee C, Link KE, Mallory M, Mann R, Maxwell M, McGraw M, McMillen D, Mukora A, Ng L, Ng L, Ngo K, Nicovich PR, Oldre A, Park D, Peng H, Penn O, Pham T, Pom A, Popović Z, Potekhina L, Rajanbabu R, Ransford S, Reid D, Rimorin C, Robertson M, Ronellenfitch K, Ruiz A, Sandman D, Smith K, Sulc J, Sunkin SM, Szafer A, Tieu M, Torkelson A, Trinh J, Tung H, Wakeman W, Ward K, Williams G, Zhou Z, Ting JT, Arkhipov A, Sümbül U, Lein ES, Koch C, Yao Z, Tasic B, Berg J, Murphy GJ, Zeng H (2020). Integrated morphoelectric and transcriptomic classification of cortical GABAergic cells. Cell.

[bib28] Granger AJ, Wallace ML, Sabatini BL (2017). Multi-transmitter neurons in the mammalian central nervous system. Current Opinion in Neurobiology.

[bib29] Hall DH, Hedgecock EM (1991). Kinesin-related gene unc-104 is required for axonal transport of synaptic vesicles in *C. elegans*. Cell.

[bib30] Hawk JD, Calvo AC, Liu P, Almoril-Porras A, Aljobeh A, Torruella-Suárez ML, Ren I, Cook N, Greenwood J, Luo L, Wang ZW, Samuel ADT, Colón-Ramos DA (2018). Integration of plasticity mechanisms within a single sensory neuron of *C. elegans* actuates a memory. Neuron.

[bib31] He S, Cuentas-Condori A, Miller DM (2019). NATF (Native and Tissue-Specific Fluorescence): A strategy for bright, tissue-specific GFP labeling of native proteins in *Caenorhabditis elegans*. Genetics.

[bib32] Huang YC, Luo J, Huang W, Baker CM, Gomes MA, Meng B, Byrne AB, Flavell SW (2023). A single neuron in *C. elegans* orchestrates multiple motor outputs through parallel modes of transmission. Current Biology.

[bib33] Jafari G, Xie Y, Kullyev A, Liang B, Sze JY (2011). Regulation of extrasynaptic 5-HT by serotonin reuptake transporter function in 5-HT-absorbing neurons underscores adaptation behavior in *Caenorhabditis elegans*. The Journal of Neuroscience.

[bib34] Juge N, Omote H, Moriyama Y (2013). Vesicular GABA transporter (VGAT) transports β-alanine. Journal of Neurochemistry.

[bib35] Jumper J, Evans R, Pritzel A, Green T, Figurnov M, Ronneberger O, Tunyasuvunakool K, Bates R, Žídek A, Potapenko A, Bridgland A, Meyer C, Kohl SAA, Ballard AJ, Cowie A, Romera-Paredes B, Nikolov S, Jain R, Adler J, Back T, Petersen S, Reiman D, Clancy E, Zielinski M, Steinegger M, Pacholska M, Berghammer T, Bodenstein S, Silver D, Vinyals O, Senior AW, Kavukcuoglu K, Kohli P, Hassabis D (2021). Highly accurate protein structure prediction with AlphaFold. Nature.

[bib36] Kamalova A, Nakagawa T (2021). AMPA receptor structure and auxiliary subunits. The Journal of Physiology.

[bib37] Koonce NL, Emerson SE, Bhaskar D, Kuchroo M, Moyle MW, Arroyo-Morales P, Martínez NV, Emerson JI, Krishnaswamy S, Mohler W, Colón-Ramos D (2024). NeuroSC: exploring neurodevelopment via spatiotemporal collation of anatomical networks. bioRxiv.

[bib38] Kumar S, Sharma AK, Leifer AM (2024). An inhibitory acetylcholine receptor gates context-dependent mechanosensory processing in *C. elegans*. iScience.

[bib39] Lacin H, Chen HM, Long X, Singer RH, Lee T, Truman JW (2019). Neurotransmitter identity is acquired in a lineage-restricted manner in the *Drosophila* CNS. eLife.

[bib40] Lee RY, Sawin ER, Chalfie M, Horvitz HR, Avery L (1999). EAT-4, a homolog of a mammalian sodium-dependent inorganic phosphate cotransporter, is necessary for glutamatergic neurotransmission in *Caenorhabditis elegans*. The Journal of Neuroscience.

[bib41] Lee S, Kim K, Zhou ZJ (2010). Role of ACh-GABA cotransmission in detecting image motion and motion direction. Neuron.

[bib42] Li HQ, Pratelli M, Godavarthi S, Zambetti S, Spitzer NC (2020). Decoding neurotransmitter switching: the road forward. The Journal of Neuroscience.

[bib43] Li H-Q, Jiang W, Ling L, Pratelli M, Chen C, Gupta V, Godavarthi SK, Spitzer NC (2024). Generalized fear after acute stress is caused by change in neuronal cotransmitter identity. Science.

[bib44] Liu C, Goel P, Kaeser PS (2021). Spatial and temporal scales of dopamine transmission. Nature Reviews. Neuroscience.

[bib45] López-Cruz A, Sordillo A, Pokala N, Liu Q, McGrath PT, Bargmann CI (2019). Parallel multimodal circuits control an innate foraging behavior. Neuron.

[bib46] Maddaloni G, Chang YJ, Senft RA, Dymecki SM (2024). Adaptation to photoperiod via dynamic neurotransmitter segregation. Nature.

[bib47] Maicas M, Jimeno-Martín Á, Millán-Trejo A, Alkema MJ, Flames N (2021). The transcription factor LAG-1/CSL plays a Notch-independent role in controlling terminal differentiation, fate maintenance, and plasticity of serotonergic chemosensory neurons. PLOS Biology.

[bib48] Marquina-Solis J, Feng L, Vandewyer E, Beets I, Hawk J, Colón-Ramos DA, Yu J, Fox BW, Schroeder FC, Bargmann CI (2024). Antagonism between neuropeptides and monoamines in a distributed circuit for pathogen avoidance. Cell Reports.

[bib49] Martens H, Weston MC, Boulland J-L, Grønborg M, Grosche J, Kacza J, Hoffmann A, Matteoli M, Takamori S, Harkany T, Chaudhry FA, Rosenmund C, Erck C, Jahn R, Härtig W (2008). Unique luminal localization of VGAT-C terminus allows for selective labeling of active cortical GABAergic synapses. The Journal of Neuroscience.

[bib50] Mathews EA, Mullen GP, Manjarrez JR, Rand JB (2015). Unusual regulation of splicing of the cholinergic locus in *Caenorhabditis elegans*. Genetics.

[bib51] McIntire SL, Jorgensen E, Kaplan J, Horvitz HR (1993). The GABAergic nervous system of *Caenorhabditis elegans*. Nature.

[bib52] McIntire SL, Reimer RJ, Schuske K, Edwards RH, Jorgensen EM (1997). Identification and characterization of the vesicular GABA transporter. Nature.

[bib53] Mclntire SL, Jorgensen E, Horvitz HR (1993). Genes required for GABA function in *Caenorhabditis elegans*. Nature.

[bib54] Morrie RD, Feller MB (2015). An asymmetric increase in inhibitory synapse number underlies the development of a direction selective circuit in the retina. The Journal of Neuroscience.

[bib55] Mullen GP, Mathews EA, Saxena P, Fields SD, McManus JR, Moulder G, Barstead RJ, Quick MW, Rand JB (2006). The *Caenorhabditis elegans* snf-11 gene encodes a sodium-dependent GABA transporter required for clearance of synaptic GABA. Molecular Biology of the Cell.

[bib56] Muñoz-Jiménez C, Ayuso C, Dobrzynska A, Torres-Mendéz A, Ruiz PC, Askjaer P (2017). An Efficient FLP-based toolkit for spatiotemporal control of gene expression in. Genetics.

[bib57] Nguyen JP, Shipley FB, Linder AN, Plummer GS, Liu M, Setru SU, Shaevitz JW, Leifer AM (2016). Whole-brain calcium imaging with cellular resolution in freely behaving *Caenorhabditis elegans*. PNAS.

[bib58] O’Malley DM, Sandell JH, Masland RH (1992). Co-release of acetylcholine and GABA by the starburst amacrine cells. The Journal of Neuroscience.

[bib59] Pei J, Grishin NV (2001). AL2CO: calculation of positional conservation in a protein sequence alignment. Bioinformatics.

[bib60] Pereira L, Kratsios P, Serrano-Saiz E, Sheftel H, Mayo AE, Hall DH, White JG, LeBoeuf B, Garcia LR, Alon U, Hobert O (2015). A cellular and regulatory map of the cholinergic nervous system of *C. elegans*. eLife.

[bib61] Pereira L, Aeschimann F, Wang C, Lawson H, Serrano-Saiz E, Portman DS, Großhans H, Hobert O (2019). Timing mechanism of sexually dimorphic nervous system differentiation. eLife.

[bib62] Pettersen EF, Goddard TD, Huang CC, Meng EC, Couch GS, Croll TI, Morris JH, Ferrin TE (2021). UCSF ChimeraX: Structure visualization for researchers, educators, and developers. Protein Science.

[bib63] Pierce-Shimomura JT, Morse TM, Lockery SR (1999). The fundamental role of pirouettes in *Caenorhabditis elegans* chemotaxis. The Journal of Neuroscience.

[bib64] Pocock R, Hobert O (2010). Hypoxia activates a latent circuit for processing gustatory information in *C. elegans*. Nature Neuroscience.

[bib65] Prevedel R, Yoon Y-G, Hoffmann M, Pak N, Wetzstein G, Kato S, Schrödel T, Raskar R, Zimmer M, Boyden ES, Vaziri A (2014). Simultaneous whole-animal 3D imaging of neuronal activity using light-field microscopy. Nature Methods.

[bib66] Reilly MB, Tekieli T, Cros C, Aguilar GR, Lao J, Toker IA, Vidal B, Leyva-Díaz E, Bhattacharya A, Cook SJ, Smith JJ, Kovacevic I, Gulez B, Fernandez RW, Bradford EF, Ramadan YH, Kratsios P, Bao Z, Hobert O (2022). Widespread employment of conserved *C. elegans* homeobox genes in neuronal identity specification. PLOS Genetics.

[bib67] Robinson RA, Stokes R (1959). Electrolyte Solutions.

[bib68] Roghani A, Feldman J, Kohan SA, Shirzadi A, Gundersen CB, Brecha N, Edwards RH (1994). Molecular cloning of a putative vesicular transporter for acetylcholine. PNAS.

[bib69] Santos MS, Park CK, Foss SM, Li H, Voglmaier SM (2013). Sorting of the vesicular GABA transporter to functional vesicle pools by an atypical dileucine-like motif. The Journal of Neuroscience.

[bib70] Sato H, Kunitomo H, Fei X, Hashimoto K, Iino Y (2021). Glutamate signaling from a single sensory neuron mediates experience-dependent bidirectional behavior in *Caenorhabditis elegans*. Cell Reports.

[bib71] Scheffer LK, Xu CS, Januszewski M, Lu Z, Takemura S-Y, Hayworth KJ, Huang GB, Shinomiya K, Maitlin-Shepard J, Berg S, Clements J, Hubbard PM, Katz WT, Umayam L, Zhao T, Ackerman D, Blakely T, Bogovic J, Dolafi T, Kainmueller D, Kawase T, Khairy KA, Leavitt L, Li PH, Lindsey L, Neubarth N, Olbris DJ, Otsuna H, Trautman ET, Ito M, Bates AS, Goldammer J, Wolff T, Svirskas R, Schlegel P, Neace E, Knecht CJ, Alvarado CX, Bailey DA, Ballinger S, Borycz JA, Canino BS, Cheatham N, Cook M, Dreher M, Duclos O, Eubanks B, Fairbanks K, Finley S, Forknall N, Francis A, Hopkins GP, Joyce EM, Kim S, Kirk NA, Kovalyak J, Lauchie SA, Lohff A, Maldonado C, Manley EA, McLin S, Mooney C, Ndama M, Ogundeyi O, Okeoma N, Ordish C, Padilla N, Patrick CM, Paterson T, Phillips EE, Phillips EM, Rampally N, Ribeiro C, Robertson MK, Rymer JT, Ryan SM, Sammons M, Scott AK, Scott AL, Shinomiya A, Smith C, Smith K, Smith NL, Sobeski MA, Suleiman A, Swift J, Takemura S, Talebi I, Tarnogorska D, Tenshaw E, Tokhi T, Walsh JJ, Yang T, Horne JA, Li F, Parekh R, Rivlin PK, Jayaraman V, Costa M, Jefferis GS, Ito K, Saalfeld S, George R, Meinertzhagen IA, Rubin GM, Hess HF, Jain V, Plaza SM (2020). A connectome and analysis of the adult *Drosophila* central brain. eLife.

[bib72] Schindelin J, Arganda-Carreras I, Frise E, Kaynig V, Longair M, Pietzsch T, Preibisch S, Rueden C, Saalfeld S, Schmid B, Tinevez J-Y, White DJ, Hartenstein V, Eliceiri K, Tomancak P, Cardona A (2012). Fiji: an open-source platform for biological-image analysis. Nature Methods.

[bib73] Schrödel T, Prevedel R, Aumayr K, Zimmer M, Vaziri A (2013). Brain-wide 3D imaging of neuronal activity in *Caenorhabditis elegans* with sculpted light. Nature Methods.

[bib74] Schwartz ML, Jorgensen EM (2016). Saptrap, a toolkit for high-throughput CRISPR/Cas9 gene modification in *Caenorhabditis elegans*. Genetics.

[bib75] Seggewisse A, Winding M (2024). Mapping the fly nerve cord. eLife.

[bib76] Serrano-Saiz E, Poole RJ, Felton T, Zhang F, De La Cruz ED, Hobert O (2013). Modular control of glutamatergic neuronal identity in *C. elegans* by distinct homeodomain proteins. Cell.

[bib77] Serrano-Saiz E, Pereira L, Gendrel M, Aghayeva U, Bhattacharya A, Howell K, Garcia LR, Hobert O (2017). A Neurotransmitter atlas of the *Caenorhabditis elegans* male nervous system reveals sexually dimorphic neurotransmitter usage. Genetics.

[bib78] Seydoux G, Fire A (1994). Soma-germline asymmetry in the distributions of embryonic RNAs in *Caenorhabditis elegans*. Development.

[bib79] Silm K, Yang J, Marcott PF, Asensio CS, Eriksen J, Guthrie DA, Newman AH, Ford CP, Edwards RH (2019). Synaptic vesicle recycling pathway determines neurotransmitter content and release properties. Neuron.

[bib80] Sitko AA, Frank MM, Romero GE, Hunt M, Goodrich LV (2025). Lateral olivocochlear neurons modulate cochlear responses to noise exposure. PNAS.

[bib81] Südhof TC (2021). The cell biology of synapse formation. The Journal of Cell Biology.

[bib82] Sulston JE, Horvitz HR (1977). Post-embryonic cell lineages of the nematode, *Caenorhabditis elegans*. Developmental Biology.

[bib83] Sulston JE, Schierenberg E, White JG, Thomson JN (1983). The embryonic cell lineage of the nematode *Caenorhabditis elegans*. Developmental Biology.

[bib84] Taylor SR, Santpere G, Weinreb A, Barrett A, Reilly MB, Xu C, Varol E, Oikonomou P, Glenwinkel L, McWhirter R, Poff A, Basavaraju M, Rafi I, Yemini E, Cook SJ, Abrams A, Vidal B, Cros C, Tavazoie S, Sestan N, Hammarlund M, Hobert O, Miller DM (2021). Molecular topography of an entire nervous system. Cell.

[bib85] Thomas M (2025). Software Heritage.

[bib86] Trudeau L-E, El Mestikawy S (2018). Glutamate cotransmission in cholinergic, GABAergic and monoamine systems: contrasts and commonalities. Frontiers in Neural Circuits.

[bib87] Uchida O, Nakano H, Koga M, Ohshima Y (2003). The *C. elegans* che-1 gene encodes a zinc finger transcription factor required for specification of the ASE chemosensory neurons. Development.

[bib88] Vaaga CE, Borisovska M, Westbrook GL (2014). Dual-transmitter neurons: functional implications of co-release and co-transmission. Current Opinion in Neurobiology.

[bib89] Wang C, Vidal B, Sural S, Loer C, Aguilar GR, Merritt DM, Toker IA, Vogt MC, Cros CC, Hobert O (2024). A neurotransmitter atlas of *C. elegans* males and hermaphrodites. eLife.

[bib90] Ward S (1973). Chemotaxis by the nematode *Caenorhabditis elegans*: identification of attractants and analysis of the response by use of mutants. PNAS.

[bib91] White JG, Southgate E, Thomson JN, Brenner S (1986). The structure of the nervous system of the nematode *Caenorhabditis elegans*. Philosophical Transactions of the Royal Society of London. Series B, Biological Sciences.

[bib92] Wu JS, Yi E, Manca M, Javaid H, Lauer AM, Glowatzki E (2020). Sound exposure dynamically induces dopamine synthesis in cholinergic LOC efferents for feedback to auditory nerve fibers. eLife.

[bib93] Wu X, Hammer JA (2021). ZEISS airyscan: optimizing usage for fast, gentle, super-resolution imaging. Methods in Molecular Biology.

[bib94] Yi Y, Li Y, Zhang S, Men Y, Wang Y, Jing D, Ding J, Zhu Q, Chen Z, Chen X, Li J-L, Wang Y, Wang J, Peng H, Zhang L, Luo W, Feng JQ, He Y, Ge W-P, Zhao H (2024). Mapping of individual sensory nerve axons from digits to spinal cord with the transparent embedding solvent system. Cell Research.

[bib95] Yu CY, Chang HC (2022). Glutamate signaling mediates *C. elegans* behavioral plasticity to pathogens. iScience.

