# Supplementary material for: SynaptoTagMe, a toolkit for in vivo mapping and modulating neurotransmission at single-cell resolution
Source: eLife. 2026 Jun 25;14:RP108675. doi: 10.7554/eLife.108675 (PMC13299628; doi:10.7554/eLife.108675)
Supplement: Peer review history

# Editor report: eLife Assessment

**DOI:** 10.7554/eLife.108675.3.sa0

**Authors:** Kavita Babu

**Affiliations:** Indian Institute of Science Bangalore India

**Keywords:** Convincing, Important

This **important** advancement in the field of neurotransmission delivers a novel toolkit for in vivo visualization of vesicular transporters for ACh, GABA, glutamate and monoamines in *C. elegans*. With the application of newly developed neuron-specific knockout methods for these vesicular transporters, the results **convincingly** demonstrate that over 10% of the neurons studied show transporter co-expression that may be correlated with co-transmission. These findings and toolkit will be of interest towards the study of neural circuit function.

# Referee report: Reviewer #1 (Public review)

**DOI:** 10.7554/eLife.108675.3.sa1

Summary:

This study presents a novel toolkit for visualizing and manipulating neurotransmitter-specific vesicles in *C. elegans* neurons, addressing the challenge of tracking neurotransmitter dynamics at the level of individual synapses. The authors engineered endogenously tagged vesicular transporters for glutamate, GABA, acetylcholine, and monoamines, enabling cell-specific labeling while maintaining physiological function. Additionally, they developed conditional knockout strains to disrupt neurotransmitter synthesis in single neurons. The study reveals that over 10% of neurons in *C. elegans* exhibit co-transmission, with a detailed case study on the ADF sensory neuron, where serotonin and acetylcholine are trafficked in distinct vesicle pools. The approach provides a powerful platform for studying neurotransmitter identity, synaptic architecture, and co-transmission.

Strengths:

(1) This toolkit offers a generalizable framework that can be applied to other model organisms, advancing the ability to investigate synaptic plasticity and neural circuit logic with molecular precision.

(2) The use of this toolkit, the authors uncover molecular heterogeneity at individual synapses, revealing co-transmission in over 10% of neurons, and offers new insights into neurotransmitter trafficking and synaptic plasticity, advancing our understanding of synaptic organization.

Weaknesses:

(1) While the article introduces valuable tools for visualizing neurotransmitter vesicles in vivo, the core techniques are based on previously established methods. The study does not present significant technological breakthroughs, limiting the novelty of the methodological advancements.

(2) The article does not fully explore the potential implications or the underlying mechanisms governing this process, while the discovery of co-transmission in over 10% of neurons is an intriguing finding. A deeper investigation into the functional uniqueness and interactions of neurotransmitters released from individual co-transmitting neurons-perhaps through case study example-would strengthen the study's impact.

Comments on revisions:

I have no further questions regarding this work. I would like to congratulate the authors on the forthcoming publication of their manuscript. This study presents a versatile methodological framework with strong potential to advance the field of neuroscience, particularly in dissecting neural circuit function and neurotransmission dynamics in vivo.

# Referee report: Reviewer #2 (Public review)

**DOI:** 10.7554/eLife.108675.3.sa2

Summary:

In this manuscript, the authors developed fluorescent reporters to visualize the subcellular localization of vesicular transporters for glutamate, GABA, acetylcholine, and monoamines in vivo. They also developed cell-specific knockout methods for these vesicular transporters. To my knowledge, this is the first comprehensive toolkit to label and ablate vesicular transporters in *C. elegans*. They carefully and strategically designed the reporters, and clearly explained the rationale behind their construct designs. Meanwhile, they used previously established functional assays to confirm that the reporters are functional. They also tested and confirmed the effect of cell-specific and pan-neuronal knockout of several of these transporters.

Strengths:

The tools developed are versatile: they generated both green and red fluorescent reporters for easy combination with other reporters; they established the method for cell-type specific KO to analyze function of the neurotransmitter in different cell types. The reagents allow visualization of specific synapses among other processes and cell bodies. In addition, they also developed a binary expression method to detect co-transmission "We reasoned that if two neurotransmitters were co-expressed in the same neuron, driving Flippase under the promoter of one transmitter would activate the conditional reporter-resulting in fluorescence-only in cells also expressing a second neurotransmitter identity". Overall, this is a versatile and valuable toolkit with well-designed and carefully validated reagents. This toolkit will likely be widely used by the *C. elegans* community.

Comments on revisions:

The authors addressed my questions in the revised manuscript.

# Referee report: Reviewer #3 (Public review)

**DOI:** 10.7554/eLife.108675.3.sa3

Summary:

Cuentas-Condori et al. generate cell-specific tools for visualizing the endogenous expression of, as well as knocking out, four different classes of neurotransmitter vesicular transporters (glutamatergic, cholinergic, gabaergic and monoaminergic) in *C. elegans*. They then use these tools in an intersectional strategy to provide evidence for the co-expression of these transporters in individual neurons, suggesting co-transmission of the associated neurotransmitters.

Strengths:

A major strength of the work is the generation of several endogenous tools that will be of use to the community. Additionally, this adds to accumulating evidence of co-transmission of different classes of neurotransmitters in the nervous system.

Another strength is the comparison to previously published single cell sequencing data and other previously published data.

Weaknesses:

Co-expression of these transporters is not in and of itself sufficient to establish neurotransmitter co-release, but this caveat is acknowledged by the authors.

Comments on revisions:

The authors have addressed all of my previous concerns.

# Author comment: Author response

**DOI:** 10.7554/eLife.108675.3.sa4

**Authors:** Andrea Cuentas-Condori, Patricia Chanabá-López, Matthew Thomas, Likui Feng, Aaron Wolfe, Peter Agoba, Matthew L Schwartz, Maximillian Brown, Margaret S Ebert, Erik Jorgensen, Cornelia I Bargmann, Daniel A Colón-Ramos

**Affiliations:** Yale University New Haven United States; Yale University New Haven United States; Yale University New Haven United States; Rockefeller University New York United States; Yale University New Haven United States; Yale University New Haven United States; University of Washington School of Medicine Seattle United States; Rockefeller University New York United States; Rockefeller University New York United States; University of Utah Salt Lake City United States; Rockefeller University New York United States; Yale University New Haven United States

The following is the authors’ response to the original reviews.

**Public Reviews:**

**Reviewer #1 (Public review):**

Summary:

This study presents a novel toolkit for visualizing and manipulating neurotransmitterspecific vesicles in *C. elegans* neurons, addressing the challenge of tracking neurotransmitter dynamics at the level of individual synapses. The authors engineered endogenously tagged vesicular transporters for glutamate, GABA, acetylcholine, and monoamines, enabling cell-specific labeling while maintaining physiological function. Additionally, they developed conditional knockout strains to disrupt neurotransmitter synthesis in single neurons. The study reveals that over 10% of neurons in *C. elegans* exhibit co-transmission, with a detailed case study on the ADF sensory neuron, where serotonin and acetylcholine are trafficked in distinct vesicle pools. The approach provides a powerful platform for studying neurotransmitter identity, synaptic architecture, and co-transmission.

Strengths:

(1) This toolkit offers a generalizable framework that can be applied to other model organisms, advancing the ability to investigate synaptic plasticity and neural circuit logic with molecular precision.

(2) Through the use of this toolkit, the authors uncover molecular heterogeneity at individual synapses, revealing co-transmission in over 10% of neurons, and offer new insights into neurotransmitter trafficking and synaptic plasticity, advancing our understanding of synaptic organization.

Weaknesses:

(1) While the article introduces valuable tools for visualizing neurotransmitter vesicles in vivo, the core techniques are based on previously established methods. The study does not present significant technological breakthroughs, limiting the novelty of the methodological advancements.

The reviewer is correct that this study does not introduce fundamentally new molecular or imaging techniques. Rather, the goal of this work is to establish a generalizable and experimentally validated framework for investigating neurotransmission in vivo at single-cell resolution. To achieve this, we deliberately integrate robust and well-established approaches, including CRISPR-based genome engineering, endogenous tagging, intersectional labeling strategies, and behavioral genetics, into a unified toolkit that enables questions that were previously difficult to address in intact animals.

The novelty of the work therefore lies not in the invention of individual technologies, but in their systematic integration, functional validation, and deployment to reveal new biological insights, such as the prevalence and spatial organization of co-transmission in vivo.

(2) The article does not fully explore the potential implications or the underlying mechanisms governing this process, while the discovery of co-transmission in over 10% of neurons is an intriguing finding. A deeper investigation into the functional uniqueness and interactions of neurotransmitters released from individual co-transmitting neurons - perhaps through case study examples - would strengthen the study's impact.

We agree with the reviewer that this study does not exhaustively explore the functional implications or mechanisms of co-transmission. The primary goal of this work is to introduce and share a validated set of strains that enable monitoring and cell-specific disruption of the major neurotransmitter systems in *C. elegans*, using molecular components that are broadly conserved across species. By establishing this toolkit, we aim to enable the mechanistic, single-cell analyses of co-transmitting neurons that extend beyond the scope of the present study but represent important next steps for the field.

**Reviewer #2 (Public review):**

Summary:

In this manuscript, the authors developed fluorescent reporters to visualize the subcellular localization of vesicular transporters for glutamate, GABA, acetylcholine, and monoamines in vivo. They also developed cell-specific knockout methods for these vesicular transporters. To my knowledge, this is the first comprehensive toolkit to label and ablate vesicular transporters in *C. elegans*. They carefully and strategically designed the reporters and clearly explained the rationale behind their construct designs. Meanwhile, they used previously established functional assays to confirm that the reporters are functional. They also tested and confirmed the effect of cell-specific and pan-neuronal knockout of several of these transporters.

Strengths:

The tools developed are versatile: they generated both green and red fluorescent reporters for easy combination with other reporters; they established the method for cell-typespecific KO to analyze the function of the neurotransmitter in different cell types. The reagents allow visualization of specific synapses among other processes and cell bodies. In addition, they also developed a binary expression method to detect co-transmission "We reasoned that if two neurotransmitters were co-expressed in the same neuron, driving Flippase under the promoter of one transmitter would activate the conditional reporter - resulting in fluorescence - only in cells also expressing a second neurotransmitter identity". Overall, this is a versatile and valuable toolkit with well-designed and carefully validated reagents. This toolkit will likely be widely used by the *C. elegans* community.

Weaknesses:

The authors evaluated the positions of fluorescent puncta by visually comparing their positions with the positions of synapses indicated by EM reconstruction. It would provide stronger supportive evidence if the authors also examined co-localization of these reporters with well-established synaptic reporters previously published by their lab, such as reporters that label presynaptic sites of AIY interneurons.

We have now included images of the synaptic vesicle marker RAB-3 in neurons like ASE (new Figure S2) and RIB (new Figure S4D). We mention in the text that the patterns observed with VGLUT/EAT-4 (in Figure 2E) and VGAT/UNC-47 (Figure 3D) are like those observed in the Rab3 images (Figure S2 and S4D, now discussed in lines 180-182 and line 244, respectively), supporting labeling of presynaptic vesicles.

Additionally, we now show that in the ADF neuron, a mutant for the conserved presynaptic kinesin KIF1A, results in the accumulation of VACh/UNC-17 and VMAT/CAT-1 in the cell soma and the elimination of the signal from the ADF axon (new Figure 7D-D’). These results are also consistent with the idea that these labeled transporters localize to synaptic vesicles that fail to be transported into the axon in the absence of a functional KIF1A/UNC-104 protein (lines 408-411).

This toolkit will likely be widely used by the *C. elegans* community. To facilitate the adoption of the approach and method by worm labs, the authors should include their plan for the dissemination of all of the reagents included in the kit, along with all of the associated information, including construct sequences and the protocols for their use.

We thank the reviewer or this suggestion, and in response we now: (1) have deposited all strains that we developed in this study to the Caenorhabditis Genetics Center, (2) have created a public website with sequences and genotyping information for each allele developed (https://www.intralab.app/research-papers/cuentas-condori_etal-2026) and(3) have named the tool kit, SynaptoTagMe, and included the name in the title and in the text. We also added the information of the public website to the main text (lines 140-142) and methods section (lines 540-542).

**Reviewer #3 (Public review):**

Summary:

Cuentas-Condori et al. generate cell-specific tools for visualizing the endogenous expression of, as well as knocking out, four different classes of neurotransmitter vesicular transporters (glutamatergic, cholinergic, GABAergic, and monoaminergic) in *C. elegans*. They then use these tools in an intersectional strategy to provide evidence for the coexpression of these transporters in individual neurons, suggesting co-transmission of the associated neurotransmitters.

Strengths:

A major strength of the work is the generation of several endogenous tools that will be of use to the community. Additionally, this adds to accumulating evidence of co-transmission of different classes of neurotransmitters in the nervous system.

Weaknesses:

A weakness of the study is a lack of comparison to previously published single-cell sequencing data. These tools are alternatively described in the manuscript as superior to the sequencing data and as validation of the sequencing data, but neither claim can be assessed without knowing how they compare and contrast to that data. It is thus not clear to what extent the conclusions of this paper are an advance over what could be determined from the sequencing data on its own. Finally, some technical considerations should be discussed as potential caveats to the robustness of their intersectional strategy for concluding that certain genes are indeed co-expressed. Overall, claims about cotransmission should be tempered by the caveats presented in the discussion, suggesting that co-expression of these transporters is not in and of itself sufficient for neurotransmitter release.

To clarify, we do not claim that our tools are superior to single-cell sequencing data. Rather, we view the characterization of neurotransmitter identity as an iterative process of discovery and validation across complementary approaches. Moreover, while this study provides an additional lens through which to examine neurotransmitter identity, its primary advance is not in redefining transmitter identity per se, but in establishing a toolkit that enables direct, in vivo monitoring and manipulation of neurotransmitter use at single-cell resolution.

We do agree on the importance of explicitly comparing our findings with prior studies. In the revised manuscript we have therefore strengthened this integration by:

(1) Revising Figure S9 and its legend to indicate the source of information for each neuron;

(2) Adding a new Table 3 summarizing neurons consistently reported to have co-transmission potential;

(3) Adding a new Table 4 listing neurons previously suggested to be co-transmitter neurons but not consistently supported across datasets;

(4) Revising the Results to clarify these comparisons (lines 372-374 and 381-383); and

(5) Incorporating this discussion into the main text (lines 482–488).

In the Discussion we also now acknowledge technical caveats of the intersectional strategy, emphasizing that co-expression of vesicular transporters indicates co-transmission potential but is not, on its own, sufficient evidence of functional co-release (lines 482–488).

**Recommendations for the authors:**

**Reviewer #1 (Recommendations for the authors):**

(1) The design of different recombination sites for the transporters is a key strength of this paper. While the authors have provided justification and validation for the chosen sites, it would be valuable to know whether alternative insertion sites were tested as controls. A comparative analysis of multiple sites would provide important insights, especially for the design of similar sites in other proteins or in mammalian systems.

Our paper lists all the sites tested for labeling each synaptic vesicle transporter. To summarize this information, we have added Table 5 in the Methods section (line 591).

(2) Given the endogenous nature of the transporter design, it would be interesting to know if the authors have observed dynamic vesicle trafficking to explain the partial overlap shown in Figure 7. A dynamic approach could better capture the potential synergism and heterogeneity of co-transmission. I recommend that the authors try time-lapse imaging to explore this dynamic process further.

We agree that dynamic imaging approaches, including time-lapse analysis of vesicle trafficking, represent an exciting avenue to further investigate the spatial and temporal organization of co-transmission. Such experiments are part of ongoing work in our laboratory and will be the focus of future studies aimed at dissecting the dynamic regulation of transmitter-specific vesicle populations *in vivo*.

(3) The paper identifies co-transmission across a significant proportion of neurons, but the functional implications and interactions of neurotransmitters released from individual cotransmitting neurons are not fully explored. A case study focusing on the uniqueness and interactions of neurotransmitter release in these neurons would provide further clarity on the biological relevance of co-transmission.

We agree with the reviewer on the importance of dissecting the functional implications of co-transmission and understanding how different neurotransmitters interact within individual co-transmitting neurons *in vivo*. The primary goal of this study is to establish and share tools that enable such investigations, and we anticipate that future work, using these reagents, will examine the functional roles of co-transmission on a neuron-by-neuron basis in the future.

(4) Minor Comments:

(a) Figure S1D: The label "eat-4" in the eat-4::GFP image appears in italics.

We have corrected this.

(b) Figure 2C: The figure legend is missing the statistical significance notation (*** p).

We have corrected this.

(c) Figure 2D: The scale bar should be labeled as 10 μm.

We have added the label.

(d) Figure S4B: The image quality could be improved for better clarity.

We have replaced the image.

(e) Figure S8: The figure legend formatting needs attention, and the scale bar is missing in Figure S8C.

We have added panel labels and the scale bar.

**Reviewer #3 (Recommendations for the authors):**

(1) A comparison of the results generated in this paper to the Cengen data (or other previously published data) would greatly strengthen the paper. Figure S7 seems to be a compilation of several different data sets, but this is very unclear if so, and there is no indication of which neurons are from which data, and whether there is any conflicting evidence (or what cutoffs were used to determine co-expression from Cengen). If there are indeed conflicting results, the ramifications should be discussed. Finally, given the caveat introduced in the discussion regarding the I2 neuron not expressing GABA synthesis or reuptake machinery, a more thorough analysis of which neurons identified here do or don't express other relevant genes may be warranted.

In the revised version, we have added Tables 3 and 4 to explicitly compare our findings with CeNGEN and prior studies. Table 3 lists neurons consistently reported across independent datasets to have co-transmission potential, while Table 4 highlights neurons that have been suggested, but not consistently supported, across studies. We now also provide explicit references for each neuron in these tables and have clarified data sources and annotations in the legend to Figure S7 (now Figure S9). These additions are intended to make points of agreement and discrepancy across datasets transparent and to better contextualize our findings within existing resources.

(2) The intersectional strategy used to identify co-expression of different transporters has some caveats that should be discussed. Specifically, removing the entire open reading frame of the eat-4 gene (as opposed to employing a T2A strategy) could potentially also remove some negative regulatory elements (for example, located within introns), leading to the inappropriate expression of the fluorescent reporter. This should at least be mentioned as a potential caveat.

We have added this caveat into the discussion section (lines 511-513).

(3) The colocalization experiments performed in Figure 7 seem to rely on the use of a transgenic allele (syb7882) that was not previously validated for functionality. This is only a problem because: (a) another allele with a constitutive mRuby in the same position (ot907) did not seem to be fully functional in the thrashing assays (Figure S4F), and thus it is at least conceivable that the differences in localization are due to the non-functional transporters being relegated to compartments destined for degradation. Validating this strain (after panneuronal Flippase expression) in the thrashing assay would dispel this concern.

We have performed thrashing assays with allele *syb7882* (UNC-17::mRuby3 GLP-on) (new Figure S6), in which we find that labeling UNC-17 with *C. elegans*-optimized mRuby3 (driven by pan-cellular Flippase) results in animals whose thrashing behavior is indistinguishable from that of wild-type animals. This result is consistent with the idea that the distinct subsynaptic localizations observed between VMAT/CAT-1 and VAChT/UNC-17 in ADF neurons arise from endogenous cellular subsynaptic organization programs.

We additionally note that allele *ot907* labels UNC-17 with mKate2, not mRuby3, and that this allele is different from wild type animals in a thrashing assay (Figure S5F). The *syb7882* allele that we generated labels UNC-17 with mRuby3 and is not different from wild type in a thrashing assay. We are unsure as to these distinct phenotypes between *ot907* and *syb7882*, but note that in addition to the use of different fluorescent proteins, each allele also employs distinct linker sequences between UNC-17 and the fluorescent protein (new Figure S6). We now explain this difference in the figure legend of Figure S5 (lines 1184-1189).

Minor comments:

(1) Is there a difference between the strains imaged in Figures 3D and S3D? If so, this is not clear. If not, why are they shown twice, and why do they look so different from each other?

We have replaced panel S3D with an endogenous RAB-3::mScarlet marker in RIB neurons to show that the localization of this synaptic vesicle marker parallels the punctated pattern of UNC-47::gfp11x3 reconstituted specifically in RIB neurons. See new panel S4D and line 244.

But to explain, GFP1-10 is expressed with an extrachromosomal array, which drives variable expression of the array and can explain the difference.

(2) Strains are alternatively denoted by their effect in the main figures, and by their allele names in the supplementary figures. This can be confusing when trying to compare data between the two figures (e.g., Figures 4C and S4F). Perhaps adding the allele names as parentheticals in the main figure might help.

We have modified the paper to include the name of the alleles used in the panels of the main figures. Additionally, we now mention the specific alleles used for the functional assays in the figure legends.

(3) To better understand the ramifications and efficiency of the cat-1 FLP-mediated removal (Figure 5E), it would be interesting to compare it directly to the ADF-specific removal of tph-1 referenced in the text.

We agree that a direct comparison between the FLP-mediated removal of *cat-1* and ADFspecific removal of *tph-1* would be informative for assessing the efficiency and functional consequences of these manipulations. These experiments represent an interesting direction for future work, and we plan to pursue such comparisons in subsequent studies.

(4) ADF seems to express very low levels of cho-1 (reuptake transporter), based on the images in Figure S8. Does it express higher levels of cha-1 (synthesis)?

We have not directly compared the relative expression levels of *cho-1* and *cha-1* in ADF neurons in this study. Such quantitative comparisons of synthesis and reuptake machinery represent an interesting direction for future work but fall beyond the scope of the present manuscript.
